# Identification of RFC4 as a potential biomarker for pan‐cancer involving prognosis, tumour immune microenvironment and drugs

**DOI:** 10.1111/jcmm.18478

**Published:** 2024-06-21

**Authors:** Lei Yu, Jing Li, Mingyang Zhang, Yu Li, Jing Bai, Pengxia Liu, Jia Yan, Changshan Wang

**Affiliations:** ^1^ School of Life Science Inner Mongolia University Hohhot China; ^2^ School of Basic medical Inner Mongolia Medical University Hohhot Inner Mongolia China

**Keywords:** bioinformatics analysis, biomarker, immunity therapy, pan‐cancer, prognosis, RFC4

## Abstract

RFC4 is required for DNA polymerase δ and DNA polymerase ε to initiate DNA template expansion. Downregulated RFC4 inhibits tumour proliferation by causing S‐phase arrest and inhibiting mitosis, resulting in the reduction of tumour cells. RFC4 has been implicated that it plays an important role in the initiation and progression of cancers, but a comprehensive analysis of the role of RFC4 in cancer has not been performed. We comprehensively analysed the expression, prognosis, methylation level, splicing level, relationship of RFC4 and immune infiltration, and pan‐cancer immunotherapy response used various databases (including TCGA, GTEx, UALCAN, Oncosplicing, TIDE, TISCH, HPA and CAMOIP), and experimented its biological function in HCC. Through pan‐cancer analysis, we found that RFC4 is significantly upregulated in most tumours. The tumour patients with high expression of RFC4 have poor prognosis. The methylation level and variable splicing level of RFC4 were abnormal in most tumours compared with the adjacent tissues. Furthermore, RFC4 was closely associated with immune cell infiltration in various cancers. RFC4 was significantly co‐expressed with immune checkpoints and other immune‐related genes. The expression of RFC4 could indicate the immunotherapy efficacy of some tumours. The RFC4 expression was associated with sensitivity to specific small molecule drugs. Cell experiments have shown that downregulated RFC4 can inhibit cell cycle and tumour cell proliferation. We conducted a systematic pan‐cancer analysis of RFC4, and the results showed that RFC4 can serve as a biomarker for cancer diagnosis and prognosis. These findings open new perspectives for precision medicine.

## 1 INTRODUCTION

RFC4 is necessary for DNA polymerase δ and DNA polymerase ε to extend primed DNA templates. The RFC family functions as clamp loaders during DNA synthesis, loading PCNA onto DNA through an ATP‐dependent process.^1^, ^2^, ^3^ During the S phase of DNA replication, RFCs activate polymerase assembly and cell cycle checkpoint control. Upon DNA damage, they activate their mismatch and excision repair mechanisms after forming complexes with PCNA. Thus, RFCs play a crucial role in DNA repair activities following DNA damage.^4^, ^5^, ^6^ These researches suggest that RFC4 may play a critical role in maintain homeostatic tumour survival and has the potential to be a promising target for cancer therapy due to its remarkable ability to regulate cell division and proliferation.^7^, ^8^, ^9^

Current research indicates that RFCs are abnormally expressed in a variety of cancers. The mRNA expression of RFC4 was significantly increased in hepatocellular carcinoma tissues, and the expression of RFC4 was closely correlated with the tumour stage of hepatocellular carcinoma patients.^10^ The elevated expression of RFC4 may contribute to the development of lung adenocarcinoma through its involvement in cell cycle and DNA replication signalling pathways.^11^ Additionally, RFC4 is involved in microtubule‐binding and histone enzyme activity, both of which play crucial roles in tumour growth. Silencing of RFC4 in various tumours, such as colon, breast and liver cancers, inhibits cells in S‐phase, preventing them from progressing to subsequent mitosis and proliferation, ultimately resulting in reduced tumour cell proliferation.^12^, ^13^, ^14^ The tumour microenvironment (TME) is crucial in the development and progression of human malignancies. Alterations in tumour cell metabolism driven by oncogenes can affect TME, which can limit the immune response and hinder cancer therapy.^15^, ^16^, ^17^ Therefore, TME is a crucial factor that influences the success of immunotherapy. Further studies are necessary to gain a better understanding of the dynamic regulatory mechanisms of stromal and immune components in TME.^18^, ^19^, ^20^

While the exact mechanism of RFC4 in tumour immunity remains mostly unexplored, it is important to fully evaluate the predictive value of RFC4 in other cancers, as well as its function in TME. Therefore, this study comprehensively analysed the relationship between RFC4 expression and the prognostic value of cancer, associated pathways, DNA methylation, variable shearing, immune microenvironment, immune response and drug sensitivity to assess the oncogenic role of RFC4 and its impact on pan‐cancer immunity and therapy using multiple databases. Furthermore, the expression levels of RFC4 in normal liver and tumour tissues were analysed, along with its impact on the prognosis of HCC. Molecular biological validation was also conducted in HCC to confirm the pro‐carcinogenic role of RFC4. In conclusion, RFC4 is a promising therapeutic target and a marker of immune infiltration and poor prognosis.

## 2 MATERIALS AND METHODS

### 2.1 RFC4 mRNA and protein expression profiles

The data were used for differential expression profiling in pan‐cancer. mRNA sequencing data for RFC4 was obtained using the Sangerbox 3.0 tool (http://vip.sangerbox.com) in 33 different types of tumour tissues and 31 normal tissues. Relevant clinical data, including survival status, and clinical and pathological stage, were also collected. The Ualcan database (http://ualcan.path.uab.edu/) was used to study RFC4 protein expression in different clinical stage types for specific cancer types. The Human Protein Atlas (HPA, http://www.proteinatlas.org) was consulted to understand the localization and protein expression levels of RFC4.

### 2.2 Survival analysis and clinical correlation analysis of RFC4

The diagnostic value of RFC4 in different types of cancer was assessed using the receiver operating characteristic (ROC) analysis method via the ‘pROC’ package. To determine the diagnostic value, the area under the curve (AUC) was calculated ranging from 0.5 to 1.0, wherein a higher AUC value indicates better diagnostic ability. The diagnostic value of RFC4 for different types of cancer was assessed using the ‘pROC’ software package and the ROC analysis method. To estimate the diagnostic value, the area under the curve (AUC) was calculated, and a higher AUC value indicates better diagnostic ability. The study examined the association between RFC4 expression and patient survival outcomes, including overall survival (OS), disease‐specific survival (DSS), disease‐free interval (DFI) and progression‐free interval (PFS), used forest plots and Kaplan–Meier curves generated by the ‘survival’ package. The hazard ratio (HR) greater than 1 (HR >1) indicates that RFC4 is a risk factor for patient survival. Conversely, an HR less than 1 (HR <1) indicates a protective effect on patients. To evaluate the relationship between RFC4 expression and clinicopathologic features in LIHC, a Sankey diagram was created using the ‘ggalluvial’ package. Univariate and multivariate Cox regression analyses were conducted in LIHC used the ‘forestplot’ package.

### 2.3 Methylation analysis and differential splicing of RFC4

The DNMIVD database (http://119.3.41.228/dnmivd/index/) was used to analyse the distribution of DNA methylation at the RFC4 locus in pan‐cancer and its impact on patient prognosis. We used the UALCAN database (https://ualcan.path.uab.edu/index.html) to analyse the methylation levels of the RFC4 promoter in different tumours. Furthermore, we utilized the oncosplicing database (http://www.oncosplicing.com/) to analyse the expression of various isoforms of RFC4 in pan‐cancer and their effect on patient prognosis.

### 2.4 Immune‐related characteristics analysis of RFC4

The study utilized the ESTIMATE algorithm, available in the Sangerbox3.0 bioinformatics tool, to derive immune and stromal scores for each tumour sample and investigate the correlation between the scores and RFC4 expression. The relationship between the RFC4 gene and immune checkpoint pathway genes, chemokines, chemokine receptors and genes of characterizing MHC‐related immune pathways was calculated using the Pearson's statistical method. Additionally, we examined the relationship between RFC4 expression and levels of immune cell infiltration, such as CD8+ T cells, the M2 subtype of tumour‐associated macrophages (M2‐TAM), myeloid‐derived suppressor cells (MDSC), cancer‐associated fibroblasts (CAF) and regulatory T (Treg) cells in various cancers using TIMER, MCPcounter and CIBERSORT. Tumour mutation burden (TMB) and Microsatellite instability (MSI) as emerging predictive biomarkers closely associated with the response to immune checkpoint inhibitor (ICI) treatment. TMB reflects the presence of somatic mutation sites in the tumour genome, resulting in the production of neoantigens and immunogenicity which in turn lead to T cell responses. TMB was calculated based on somatic data obtained from the TCGA database, and the MSI score of each cancer was analysed. Then, we analysed the correlation between RFC4 expression and TMB or MSI through pearson's correlation analysis.

### 2.5 The relationship between small molecule drugs and the value of RFC4 in immunotherapy

We analysed the effect of RFC4 expression levels on the clinical prognosis of cancer patients after small molecule drugs and immunotherapy using the ‘Kaplan–Meier Immunotherapy’ module of the Kaplan–Meier Plotter (http://kmplot.com/analysis/) database. The Tumour Immune Dysfunction and Rejection Database (TIDE, http://tide.dfci.harvard.edu) collected transcriptomic data from multiple groups of immunotherapy patients. A *t*‐test was used to detect changes in RFC4 expression between the responding and non‐responding groups.

### 2.6 Analysis of RFC4 expression in scRNA‐seq datasets

To validate the predictive value of RFC4, we collected transcriptomic data and clinical information from the TISCH databases (http://tisch.comp‐genomics.org/home/) from various tumour scRNA‐Seq cohorts. These cohorts received different immunotherapies, including anti‐PD‐1 therapy, anti‐PD‐L1 therapy, anti‐CTLA4 therapy and anti‐PD‐L1 plus anti‐CTLA‐4 combination therapy. We analysed the expression of RFC4 and its correlation with immune cells under different immunotherapies, utilizing Seurat for visualization.

### 2.7 Functional analysis of RFC4 in hepatocellular carcinoma

The KEGG enrichment pathway of RFC4 in individual tumours from the CAMOIP (http://www.camoip.net/) online database was evaluated using the GSEA method. And the association of RFC4 expression with gene mutations, metabolism‐related pathways, immunoinflammatory scores and clinical factors were evaluated in LIHC set.

### 2.8 RFC4 analysis of HCC in HCCDB database

We collected three HCC gene expression profiling datasets in the HCCDB database (http://lifeome.net/database/hccdb/home.html), including GSE36376, GSE22058 and ICGC‐ LIRI‐JP. GSE36376 included 193 normal and 240 hepatocellular carcinoma tissue specimens. GSE22058 contained 97 normal and 100 HCC tissues. ICGC‐LIRI‐JP contained 177 normal and 212 HCC tissues. The differential expression of RFC4 in hepatocellular carcinoma was assessed based on these three sets of data.

### 2.9 Cell culture and transfection

HepG2 cell lines were obtained from the Type Culture Collection of the Chinese Academy of Sciences (Shanghai, China). These cells were cultured in Dulbecco's modified eagle medium (DMEM) (Gibco, USA) containing 10% fetal bovine serum (FBS; Gibco, USA), 10 U/mL penicillin and 10 mg/mL streptomycin (Sigma, USA). The cells were grown in a sterile incubator with a humidified atmosphere containing 5% CO_2_ at 37°C. A specific short hairpin RNA (shRNA) targeted to RFC4 was synthesized by Gene Pharma (Shanghai, China). The empty vector was utilized as a negative control. Lipofectamine 2000 (Thermo Fisher Scientific, Waltham, USA) was used for the transfection of all these vectors and reagents into cells.

### 2.10 Cell viability assays

Cell proliferation was evaluated by the MTS assay. Approximately 2 × 105 transfected cells per well were seeded into 96‐well plates and cultured at 37°C. After incubation for 0, 24, 48, 72 and 96 h, MTS reagent (20 μL) was added, followed by further incubation for 4 h. The absorbance was determined at 490 nm. Each independent experiment was replicated at least three times. Plasmid Construction and Cell Transfection.

### 2.11 Cell cycle assay

Clean cells with precooled PBS, digest with trypsin, and collect. Discard supernatant by centrifugation and resuspend in 500 μL of 70% precooled ethanol. Fix for 2 h or overnight. After fixation, centrifuge and resuspend in PBS for cleaning. Filter the cells through a sieve to disperse them, and then centrifuge again to precipitate the cells. Add the appropriate amount of RNaseA (100 μL for a medium dish and 200 μL for a large vessel) to the cell precipitate. Resuspend the mixture and incubate it in 37°C water bath for 30 min, avoiding exposure to light. Then, add 400 μL of PI staining solution to a centrifuge tube and incubate it on ice in the dark for 30 min before final detection.

### 2.12 Transwell assay

Human HepG2 cells were inoculated into the upper chamber with a serum‐free medium. Each well was a density of 2 × 106 cells. The bottom chamber is filled with 500 μL of 20% fetal bovine serum (FBS) culture medium. After incubating in a 5% (v/v) CO_2_ incubator at RT for 2 days, removing the non‐invasive cells and matrigel in the upper chamber, and then the cells were fixed on the lower surface with 10% neutral buffered formalin solution and 0.1% crystal violet staining. Count invading cells in five randomly selected microscope fields.

### 2.13 Statistical analysis

The Student's *t*‐test was used to compare gene expression levels in normal and cancerous tissues. Comparisons within the cell experiment were performed using Student's *t*‐test using GraphPad Prism Software (GraphPad Inc., California, USA). Statistical significance was set at *p* < 0.05. Kaplan–Meier (KM) survival curves were used to assess the prognostic significance of the analysed indexes. Cox proportional risk regression models were used to calculate adjusted risk ratios. A statistically significant result was defined as having a significance level of *p* < 0.05.

## 3 RESULTS

### 3.1 Gene expression analysis of RFC4 in pan‐cancer

**FIGURE 1:**
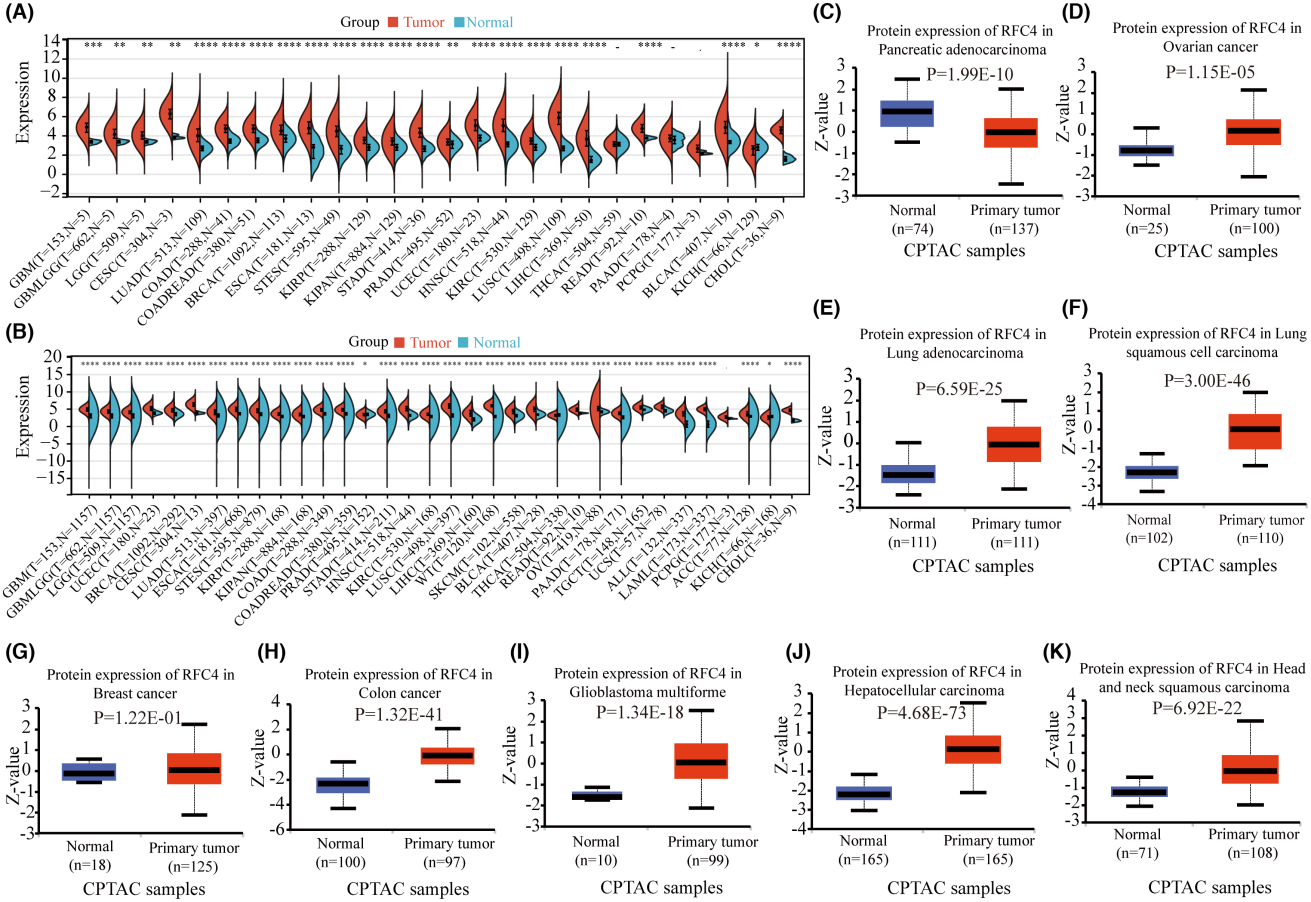
Aberrant expression levels of RFC4 in pan‐cancer. (A) Expression analysis of RFC4 mRNA from 34 types of tumour and normal tissues. (B) Combining TCGA and GTEx databases to obtain RFC4 mRNA expression levels. (C–K). RFC4 total protein levels between pancreatic adenocarcinoma, ovarian cancer, lung adenocarcinoma, lung squamous cell carcinoma, breast cancer, colon cancer, gllioblastomsa mulltifornme, hepatocelleular carcinoma, head and neck squamous carcinoma and normal tissues were shown by CPTAC. *p < 0.05; **p < 0.01; ***p < 0.001; ****p < 0.0001.

Table 1 contains abbreviations of 33 tumours from the TCGA database. We analysed RFC4 mRNA expression in 33 tumours from the TCGA database and found that RFC4 was significantly decreased in 23 types of cancers, including GBM, GBMLGB, LGG, CESC, LUAD, COAD, COADREAD, BRCA, ESCA, STES, KIRP, KIPAN, STAD, PRAD, UCEC, HNSC, KIRC, LUSC, LIHC, READ, PCPG, BLCA, KICH and CHOL (Figure 1A). In addition, due to the limited number of normal samples in the TCGA database, we combined the TCGA data with the GTEx database to assess RFC4 expression in 33 cancer types. The results showed that RFC4 was differentially expressed in 29 cancer types (Figure 1B). We analysed RFC4 protein expression levels in various cancers in the CPTAC database. The results showed that RFC4 protein expression was upregulated in COAD, GBM, LIHC, LUAD, LUSC, HNSC, PAAD and OV compared to surrounding normal tissues (*p* < 0.05, Figure 1C). Next, we analysed the correlation between RFC4 expression level and pathological stage and gender, from which we found that, stage staging of BRCA, KICH, KIRP, KIRC, LIHC, OV, ACC and KICH correlated with RFC4 expression (*p* < 0.05, Figure S1A). While T‐staging of CESC, BRCA, KIRP, KIPAN, PRAD, KIRC, LIHC, PAAD and ACC was associated with RFC4 expression (*p* < 0.05, Figure S1B), and G‐stage of GBMLGB, LGG, STES, UCEC, HNSC, LIHC, PAAD and CHOL was associated with RFC4 expression (*p* < 0.05, Figure S1C), from which we found higher expression for male in SARC and KIRP, and conversely, for female in HNSC (*p* < 0.05, Figure S1D).

**TABLE 1:** Abbreviations from the TCGA database for 33 cancers.

Cancer types	Abbreviations
Adrenocortical carcinoma	ACC
Bladder urothelial carcinoma	BLCA
Breast invasive carcinoma	BRCA
Cervical squamous cell carcinoma and endocervical adenocarcinoma	CESC
Cholangiocarcinoma	CHOL
Colon adenocarcinoma	COAD
Lymphoid neoplasm diffuse large B‐cell lymphoma	DLBC
Oesophageal carcinoma	ESCA
Glioblastoma multiforme	GBM
Head and neck squamous cell carcinoma	HNSC
Kidney chromophobe	KICH
Kidney renal clear cell carcinoma	KIRC
Kidney renal papillary cell carcinoma	KIRP
Acute myeloid leukaemia	LAML
Brain lower grade glioma	LGG
Liver hepatocellular carcinoma	LIHC
Lung adenocarcinoma	LUAD
Lung squamous cell carcinoma	LUSC
Mesothelioma	MESO
Ovarian serous cystadenocarcinoma	OV
Pancreatic adenocarcinoma	PAAD
Pheochromocytoma and paraganglioma	PCPG
Prostate adenocarcinoma	PRAD
Rectum adenocarcinoma	READ
Sarcoma	SARC
Skin cutaneous melanoma	SKCM
Stomach adenocarcinoma	STAD
Testicular germ cell tumours	TGCT
Thyroid carcinoma	THCA
Thymoma	THYM
Uterine corpus endometrial carcinoma	UCEC
Uterine carcinosarcoma	UCS
Uveal Melanoma	UVM

### 3.2 Identification of the diagnostic and prognostic value of RFC4 in pan‐cancer

AUC has sensitivity and specificity and is often used to indicate the intrinsic validity of a disease diagnosis. The results of the analysis showed that RFC4 predicted the diagnosis of nine cancer types with high accuracy (AUC > 0.9) based on the AUC values in the ROC curve, such as GBM (AUC = 0.966), LUAD (AUC = 0.959), CESC (AUC = 0.979), COAD (AUC = 0.945), LIHC (AUC = 0.971), ESCA (AUC = 0.964), LUSC (AUC = 0.998), STAD (AUC = 0.973) and HNSC (AUC = 0.967) (Figure S2). We further investigated the prognostic impact of RFC4 on cancer patients by Cox proportional risk modelling, including OS, DSS, DFI and PFI analysis. As shown in the forest plot, high RFC4 expression was a risk factor for patients and significantly predicted prognostic outcome in GBMLGG, KIPAN, ACC, LIHC, KICH, KIRC, LGG, KIRP, PAAD, LUAD, PCPG, PRAD and MESO patients, while it had a protective effect on patients in DLBC, OV and CESC (Figure S3A). The results of DSS analysis showed that RFC4 exerted a risk effect in GBMLGG, KIPAN, KIRC, KIRP, KICH, ACC, LIHC, LGG, LUAD, PRAD, MESO, PAAD, PCPG, SKCM‐P and THCA patients, whereas it had a protective effect on patients in DLBC and OV (Figure S3B). The results of DFI analysis confirmed that high RFC4 expression was a risk factor in patients with KIRP, KIPAN, LIHC, SARC, COAD and PRAD (Figure S3C). For PFS analysis, high RFC4 expression was a risk factor for patients with KIPAN, GBMLGG, KIRP, PRAD, ACC, KICH, KIRC, LIHC, LGG, SKCM‐P, SARC, PAAD, MESO and PCPG (Figure S3D). In summary, higher RFC4 expression was strongly associated with and LIHC, COAD and KIRC tumour prognosis. In summary, the AUC and prognostic analysis results indicate that RFC4 has high diagnostic and prognostic value for various types of cancer.

### 3.3 Analysis of RFC4 DNA methylation in pan‐cancer

**FIGURE 2:**
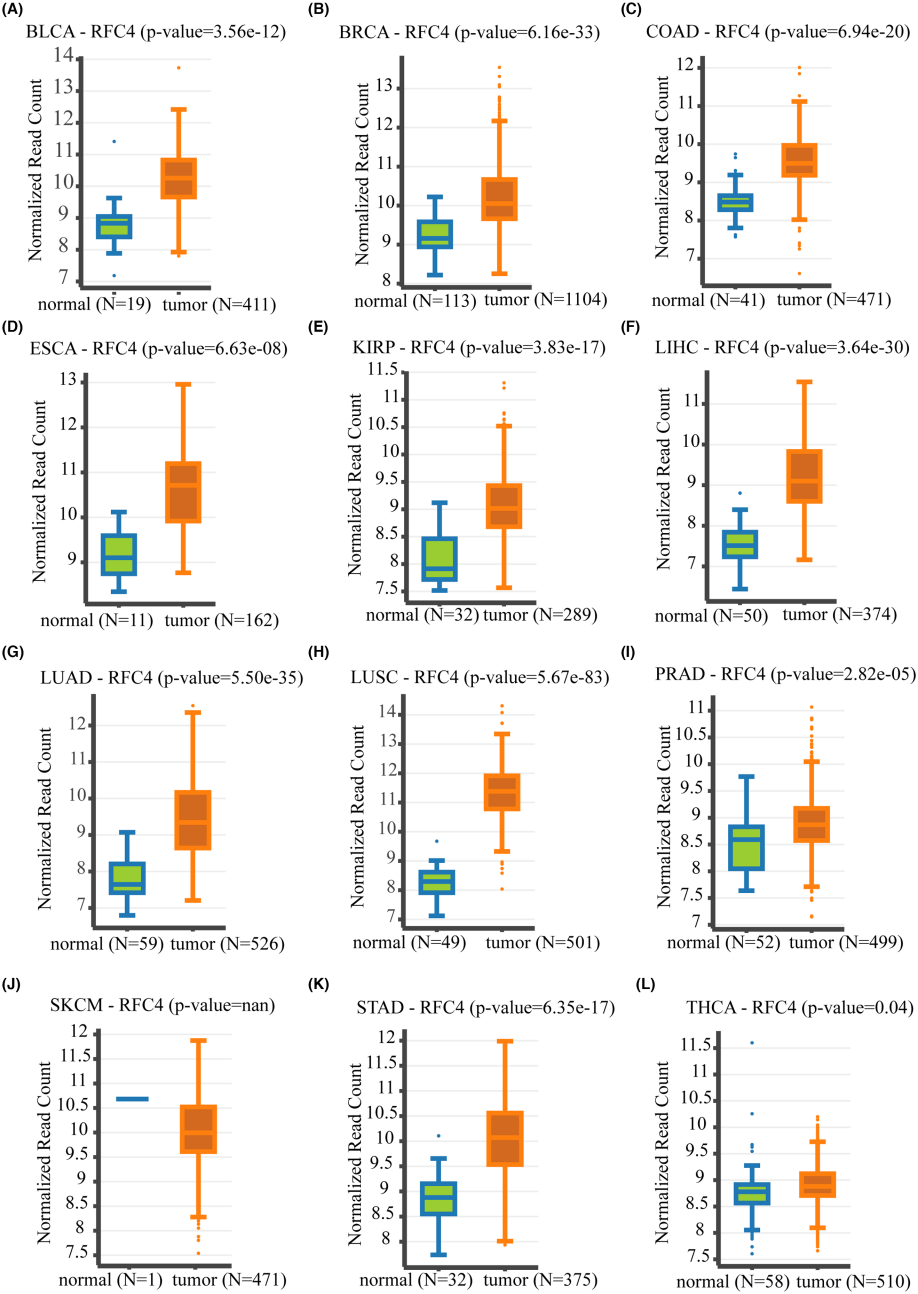
Correlation between DNA methylation levels and RFC4 expression levels in pan‐cancer. (A) BLCA, (B) BRCA, (C) COAD, (D) ESCA, (E) KIRP, (F) LIHC, (G) LUAD, (H) LUSC, (I) PRAD, (J) SKCM, (K) STAD and (L) THCA.

DNA methylation is critical for disease pathogenesis, and aberrant methylation is considered to be one of the hallmarks of cancer. We investigated the expression and prognostic levels of DNA methylation of RFC4 in tumours using the DNMIVD database. The results showed that the methylation level of RFC4 was negatively correlated with the expression of RFC4 in tumours such as BLCA, BRCA, LIHC, LUAD and STAD (*p* < 0.05, Figure 2). We counted the prognostic effects of methylation at different loci of RFC4 in 24 tumours, and divided the tumour samples by the median methylation expression level. The cg03434872 and cg08440162 were found to be prognostic protective factors in LIHC, while cg12583908 and cg09713000 were prognostic risk factors in LIHC. The cg12583908 was a protective factor for prognosis in LGG, while cg12820191 was a risk factor for LGG. The cg00373256 was a protective factor for UVM, while cg03434872 was a risk factor for UVM (Table S1). Subsequently, we analysed the methylation level of the RFC4 promoter used the UALCAN database. Compared with normal tissues, BRCA, BLCA, UCEC, LUSC, LUAD, LUSC, PRAD, HNSC, KIRP and TGCT cancer tissues had lower levels of RFC4 promoter methylation. In PAAD, SARC and KIRC, cancer tissues have higher levels of methylation than normal tissues (*p* < 0.05, Figure S4).

### 3.4 Pan‐cancer analysis of RFC4 differential splicing

RNA variable splicing is an important mechanism for regulating protein expression in eukaryotic cells, and both variable splicing and tumorigenesis are closely related. We used the SpliceSeq module from the OncoSplitting database to investigate the expression and prognostic value of three variable splices of RFC4 in tumours. RFC4_ES_68062 was significantly higher expressed in tumours including HNSC, BRCA, LUSC, KICH, STAD and ECAS were significantly higher expressed in tumours including ACC, LIHC, LAML and DLBC; Based on OS and PFI, RFC4_ES_68062 was a prognostic risk factor in ACC and CHOL (HR >1, *p* < 0.05, Figure S5). While RFC4_AA_68063 was significantly higher expressed in LUSC and STAD, and was a prognostic risk factor in KIPR and BRCA (HR >1, *p* < 0.05, Figure S6). RFC4_AA_68064 was significantly higher expressed in tumours including KIRC, COAD, THCA and KIRP, and was a prognostic risk factor based on OS analysis in GBM, ACC and DLBC and other tumours, and for PFI, RFC4_AA_68064 was a prognostic risk factor in THCA, MESO and TGCT tumours (HR >1, *p* < 0.05, Figure S7).

### 3.5 Immune‐related characteristics of RFC4 in pan‐cancer

**FIGURE 3:**
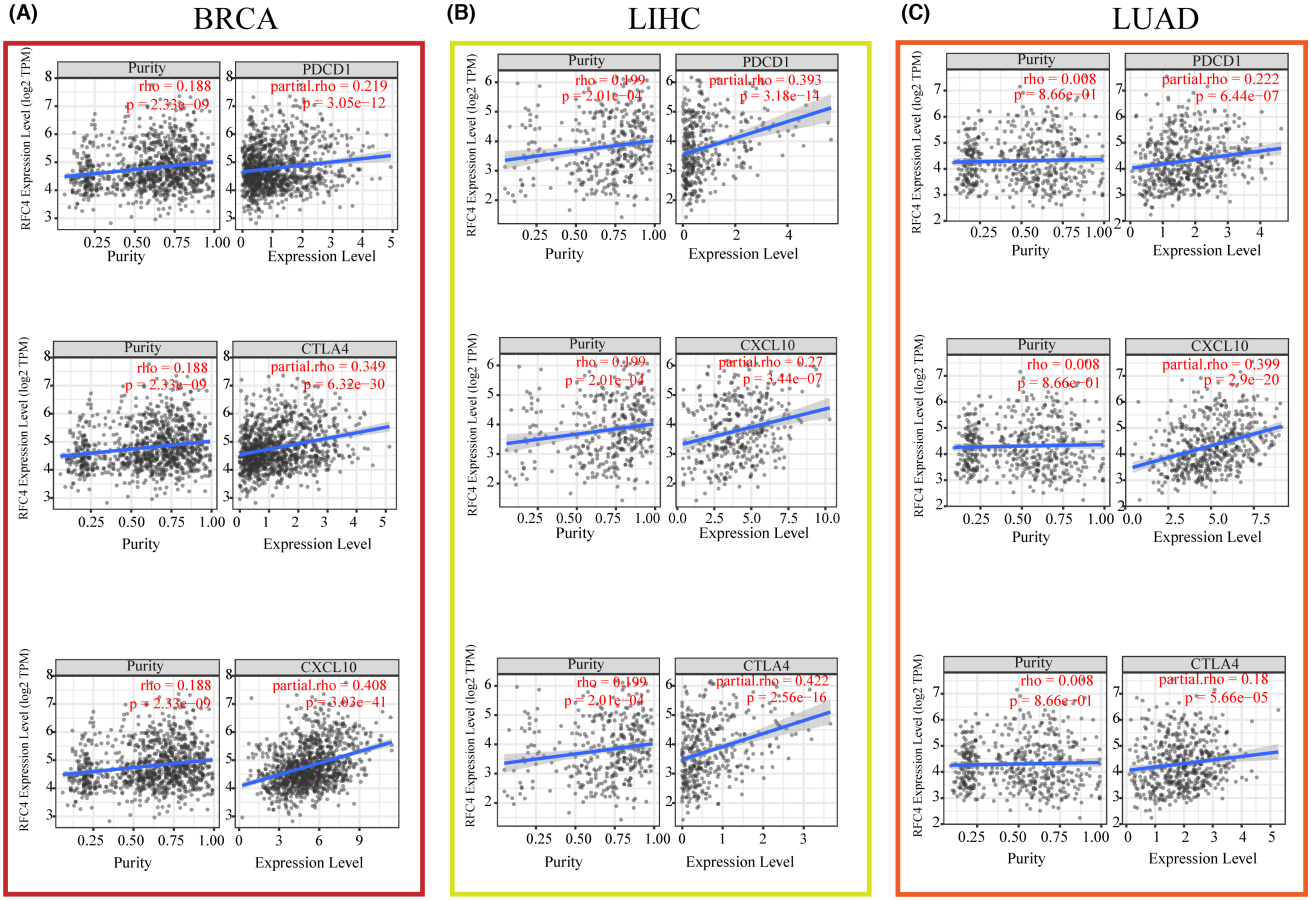
The effect of RFC4 expression on tumour immunity (A) The correlation between RFC4 and tumour purity, PDCD1, CXCL10 and CTLA4 in BRCA. (B) The correlation between RFC4 and tumour purity, PDCD1, CXCL10 and CTLA4 in LIHC. (C) The correlation between RFC4 and tumour purity, PDCD1, CXCL10 and CTLA4 in LUAD. *p < 0.05; **p < 0.01; ***p < 0.001; ****p < 0.0001.

The tumour immune microenvironment is one of the key factors influencing the outcome of tumour immunotherapy. We used the TCGA database to determine whether there is a relationship between RFC4 expression and the expression of immune‐related genes. Correlation analysis between RFC4 and checkpoint gene expression in different types of cancers showed a high correlation with immune genes including CCL4, CCL16, HLA family genes, TNFRSF8, TNFRSF14, TNFRSF18, CD70, CD44 (*p* < 0.05, Figure S8), CD276, CX3CL1 and VGEGFA (*p* < 0.05, Figure S9). In addition, significant co‐expression of RFC4 with immune checkpoint genes including PDCD1, CXCL10 and CTLA4 was found in BRCA, LIHC and LUAD (Figure 3A–C). These findings suggest that RFC4 regulates tumour immune responses through immune checkpoints. We next determined the relationship between RFC4 expression and immune cell infiltration based on the immunity scores. We found a significant correlation between RFC4 expression and immune scores such as GBM, LUAD, THYM, ESCA, LUSC and KIRP (Figure S10). In addition, we analysed the level of immune cell infiltration in different cancers using three algorithms, TIMER, MCPCOUNTER and CIBERSORT. Significant positive correlations between RFC4 expression and CD8+ T‐cell infiltration was observed in THYM, PRAD, THCA, KIRC, PAAD, PCPG, BLCA, KICH, GBM, ACC, SKCM‐P and GBMLGG; Furthermore, the expression of RFC4 is significantly positively correlated with neutrophil abundance in PRAD, THCA, KIRC, LUAD, LIHC, PAAD, BLCA, OV, SKCM‐M, SKCM‐P, SKCM and BRCA, and negatively correlated in LUSC, MESO, TGCT, SARC and GBM; Significant positive correlations between RFC4 expression and macrophage infiltration were observed in PRAD, THCA, KIRC, LIHC, BLCA, OV, KICH in TIMER algorithm (Figure S11A). Based on the MCPCOUNTER algorithm analysis, we found that RFC4 expression was significantly positively correlated with CD8 + T cell levels in THYM, LIHC, ACC, STAD, THCA, DLBC, HNSC, OV, LUAD, BLCA and UCS; In GBNLGG, ACC, THCA, PAAD, KIRC, KIPAN and KICH, RFC4 expression is significantly positively correlated with fibroblast levels, while in THYM, STES, STAD, LUSC, UVM, BRCA, HNSC, OV, COAD and COADREAD tumours, RFC4 expression is significantly negatively correlated with fibroblast levels; In TGCT, ACC, STES, ESCA, STAD, THCA, SKCM, LUSC, PAAD, SKCM‐M, KIRP, KIRC, KIPAN, SKCM‐P, OV, BLCA, KICH and PCPG, RFC4 expression is significantly positively correlated with neutrophil levels, while in THYM and LUAD, RFC4 expression is significantly negatively correlated with neutrophil levels (Figure S11B). Based on the MCPCOUNTER algorithm analysis, in 12 cancers included BLCA, PRAD, LUSC, SKCM and COAD; RFC4 expression was significantly correlated with the level of CD8+ T cells, and in 17 cancers including STES, ESCA, DLBC, THYM, UVM, STAD, BLCA and LUSC; The RFC4 expression was significantly correlated with the level of Treg cells; In 17 tumours, RFC4 expression was significantly correlated with M0 macrophage levels; The RFC4 expression was significantly correlated with M2 macrophage levels in 11 types of cancers, including KIPAN, THYM, KICH, TGCT, LGG, SKCM, GBMLGG, KIRP, ACC and LUAD; In 18 types of cancers, RFC4 expression was significantly correlated with M1 macrophage levels, and 10 cancers, including LIHC, were significantly correlated with neutrophils were significantly correlated (Figure S11C).

### 3.6 Single‐cell expression levels of RFC4 in multiple cancer tissues

**FIGURE 4:**
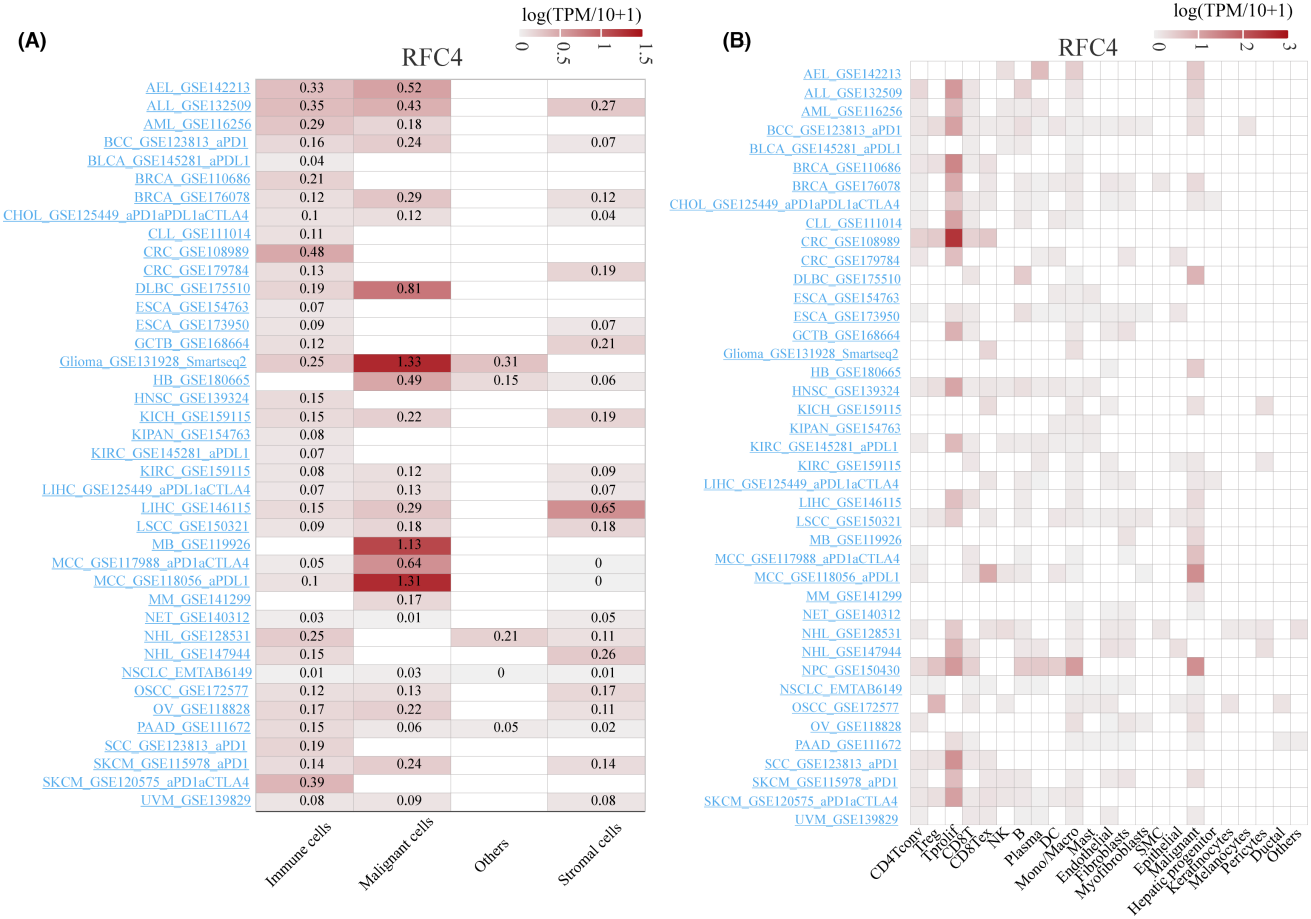
Single‐cell expression analysis of RFC4 in tumour tissues. (A) Cluster heatmaps showing the mRNA levels of RFC4 in four cell types in tumour tissues. (B) Cluster heatmaps showing the mRNA levels of RFC4 in 23 cell types in tumour tissues.

To further investigate the expression of RFC4 in various cell types of tumour tissues, we analysed the single‐cell expression of RFC4 used 40 datasets from the TISCH database. As shown in Figure 4A, the heatmap reveals a high expression level of RFC4 in immune and tumour cells, indicating a wide range of RFC4 expression in various immune and tumour cells, and the heatmap revealed the relative expression levels of RFC4 in 23 cell types, indicating a wide range of RFC4 expression in a variety of immunities and cells (Figure 4B). UMAP plots revealed the expression of RFC4 in T cell, B cell and macrophages in basal cell carcinoma (BCC_GSE123813_aPD1), with particularly high levels of RFC4 expression observed in proliferating T cells (Tprolif) where particularly high expression was observed (Figure S12A). In LIHC (LIHC_GSE140228_10X), RFC4 was predominantly expressed in Tprolif and malignant cells (Figure S12B). Interestingly, in SCC (scC_GSE123813_aPD1, Figure S12C), RFC4 was predominantly expressed in Tprolif. Moreover, RFC4 showed similar expression patterns between Tprolif and macrophages in multiple cancer tissues, suggesting its potential regulatory role in T cell function.

### 3.7 Relationship between RFC4 and immunotherapy‐related traits in pan‐cancer

**FIGURE 5:**
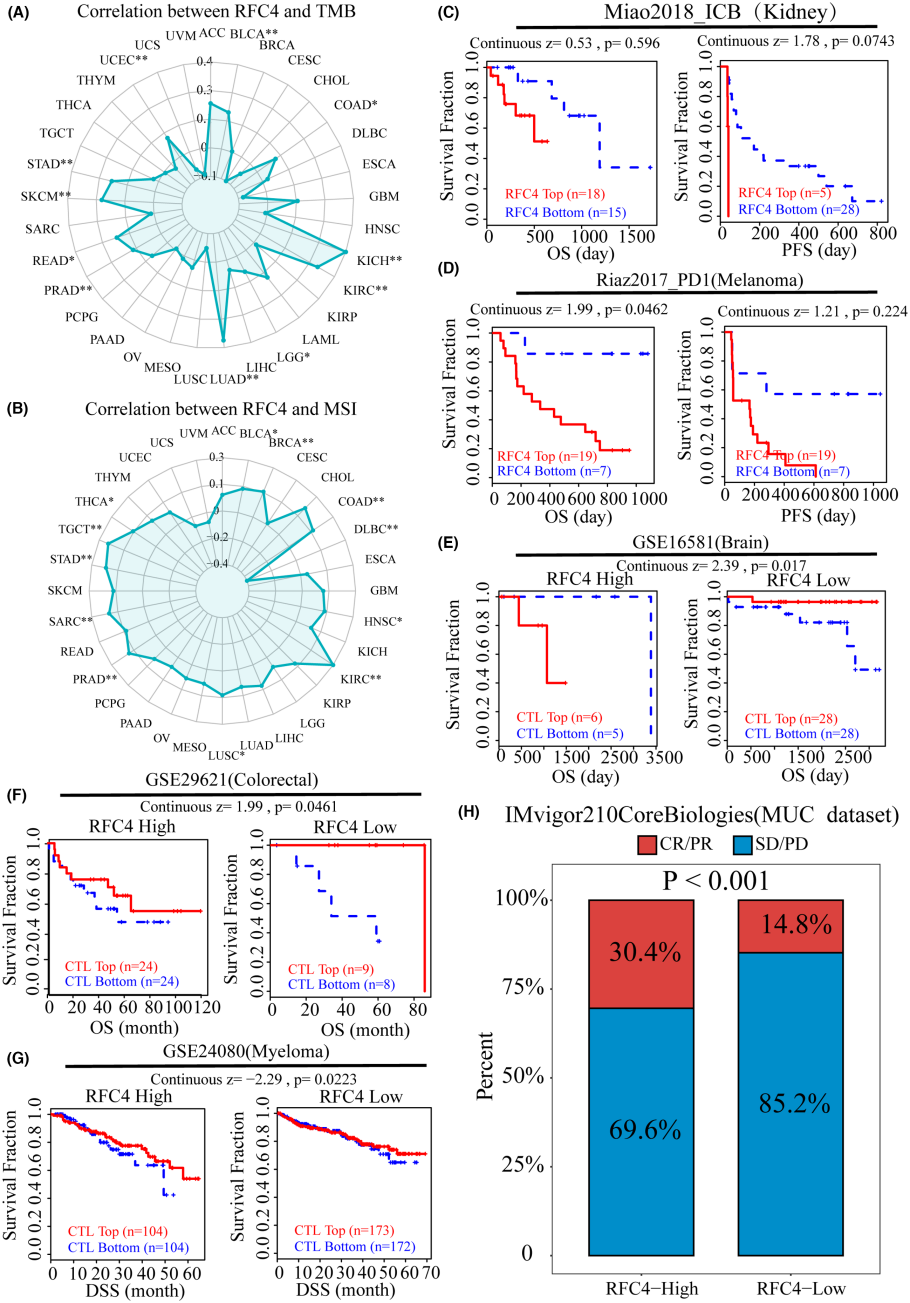
(A) The radar image shows the correlation between RFC4 expression and tumour mutation burden (TMB). (B) The correlation between RFC4 expression and microsatellite instability (MSI). (C–D) RFC4 expression predicts OS and PFS in renal cancer and melanoma patients undergoing PD‐1/PD‐L1 immunotherapy. (E–G) RFC4 expression predicts OS in immunotherapy for patients with brain cancer (GSE16581), colon cancer (GSE29621) and myeloma (GSE24080). (H) Response rate of BLCA patients to immunotherapy. PD refers to disease progression; SD refers to stable disease; CR refers to complete response, PR refers to partial response. *p < 0.05; **p < 0.01; ***p < 0.001; ****p < 0.0001.

Cancer immunotherapy has made significant progress through immune checkpoint inhibitors (ICIs) therapy. However, in most cancer types, only a small percentage of patients respond to ICIs therapy. Therefore, the search for new markers of immunotherapeutic response is crucial for cancer immunotherapy. TMB can indirectly reflect the ability and degree of neoantigen production by tumours and predict the efficacy of immunotherapy for a wide range of tumours, and MSI can also predict the immunotherapy of tumours. Therefore, we analysed RFC4 of expression correlated with TMB and MSI in cancer, and the expression of RFC4 was positively correlated with TMB in BLCA, COAD, KICH, KIRC, LGG, LUAD, PRAD, READ, SKCM, STAD and UCEC (Figure 5A). In BLCA, BRCA, COAD, HNSC, KIRC, LUSC, PRAD, SARC, STAD, TGCT and THCA, RFC4 expression was positively correlated with MSI, but negatively correlated in DLBC (Figure 5B). Next, we explored the relationship between ICB treatment response and RFC4 using the TIDE database. According to the Miao2018_ICB and Riaz2017_PD1 cohorts, high RFC4 expression affected PD‐1 blockade and decreased OS and PFS in KIRC and SKCM patients (Figure 5C,D). On the contrary, the GSE16581 cohort, GSE29621 cohort and GSE24080 cohort showed that when RFC4 expression was elevated, tumour patients would experience longer OS and DSS after ICI treatment (Figure 5E–G), and we found that the UVM immunotherapy cohort in the IMvigor210CoreBiologies using the RFC4 high expression group had a higher immune response than the low‐expressing group (*p* < 0.001, Figure 5H). We also predicted the prognostic value and efficacy of RFC4 in the immunotherapy cohort using the Kaplan–Meier Plotter website, and we found that the AUC values of RFC4 exceeded 0.5 in all of the anti‐PD‐1 treatment cohorts, with high expression of RFC4 being a protective factor in the Bladder and Melanoma samples, and the high RFC4 expression was a risk factor in Glioblastoma samples, but there was difference in RFC4 expression between the drug‐responsive and non‐responsive groups in the three tumour samples (Figure S13A–C). Interestingly, the AUC value of RFC4 in the anti‐PD‐L1 treatment cohort also exceeded 0.5, and RFC4 was also predictive of immunotherapy outcome in patients treated with PD‐L1 (Figure S13D,E). Overall, aberrant RFC4 expression can guide and benefit clinical therapy.

### 3.8 RFC4 affects cancer therapeutic responses and the molecular docking of RFC4‐targeted compounds

**FIGURE 6:**
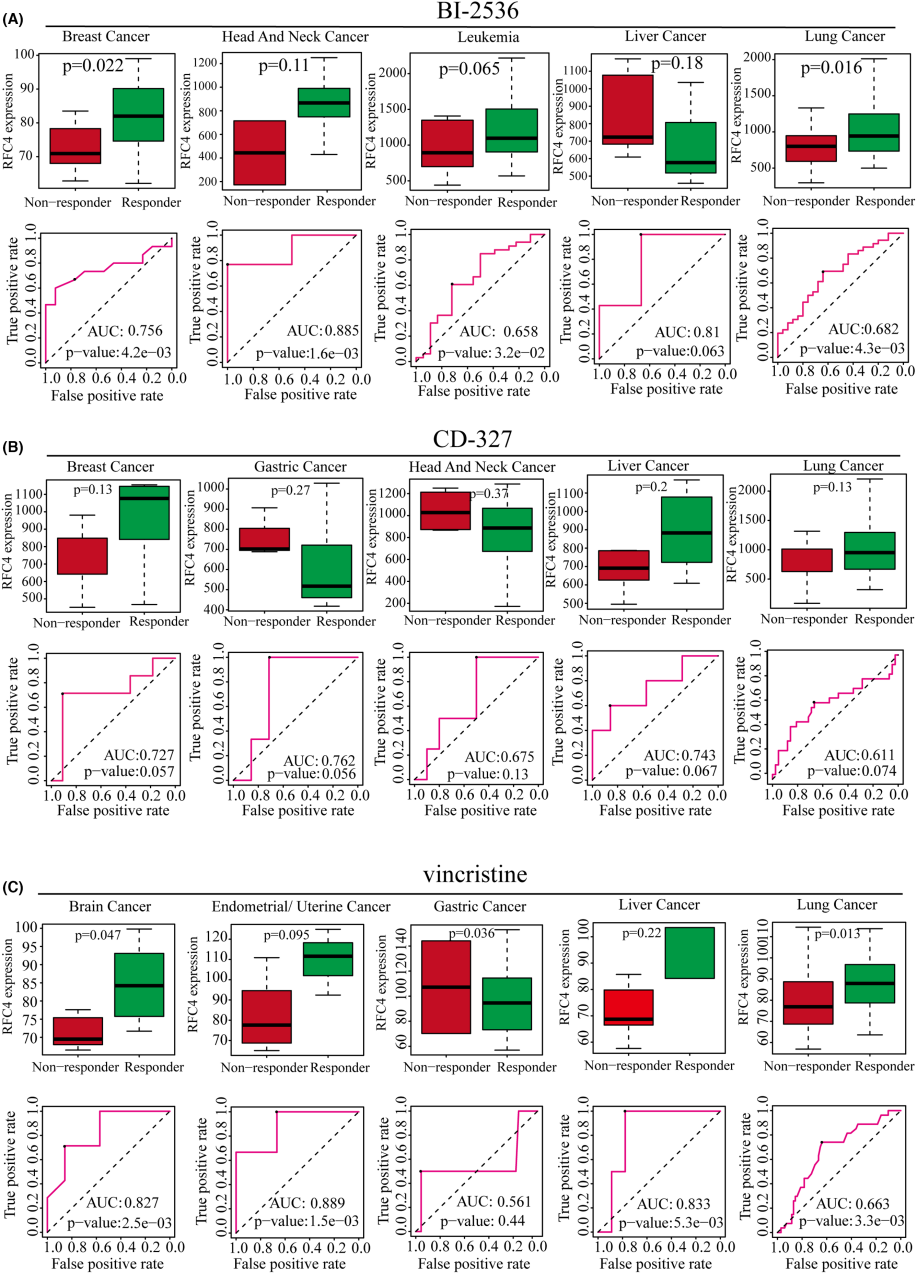
Response and drug sensitivity analysis of RFC4 to chemotherapy. (A) The relationship between RFC4 expression level and BI‐2536 in five types of cancer, (B) the relationship between RFC4 expression level and CD‐327 in five types of cancer, and (C) the relationship between RFC4 expression level and vincristine in five types of cancer. The ROC curve reflects the predictive effect of RFC4 levels on chemotherapy response.

To investigate whether RFC4 predicts cancer treatment response, we obtained the relationship between different small molecule therapeutic treatments and RFC4 expression from the ROC mapper. We found that RFC4 was highly expressed in responders in the breast cancer cohort after BI‐2536 treatment, and the AUC value of OS after BI‐2536 treatment reached 0.756. In the remaining four tumour treatments, RFC4 expression was also upregulated to varying degrees in responders, and the BI‐2536 treatment OS of AUC values all exceeded 0.6 (Figure 6A). After CD‐327 treatment, RFC4 was highly expressed in non‐responders in breast, liver and lung cancer patients, and in RFC4 responders in gastric and head and neck cancer patients, but the AUC values of OS exceeded 0.6 (Figure 6B). After vincristine treatment, RFC4 was highly expressed in brain, endometrial, liver and lung cancers in non‐responders and in gastric and RFC4 responders, and the AUC values of OS exceeded 0.6 (Figure 6C). In addition, we also evaluated the predictive value of RFC4 in afatinib‐, GSK461364‐ and SB‐225002‐treated patients, respectively. In the afatinib‐treated cohort, RFC4 was highly expressed in non‐responders in breast cancer and leukaemia patients, and in head and neck, liver and gastric cancers and in mid‐RFC4 responders; however, the area under the curve (AUC) values of OS were higher than 0.6 in all cohorts. area under the curve (AUC) values exceeded 0.5 in all cohorts. In GSK461364, SB‐225002‐treated patients, non‐responders in breast, head and neck, and hepatocellular carcinoma patients were characterized by high expression of RFC4, and the area under the curve (AUC) values of OS exceeded 0.5 in all cohorts (Figure S14). To validate the substantial relationship between RFC4 and small molecule drugs next, we chose BI‐ 2536, afatinib and vincristine for molecular docking analysis. The results showed that BI‐2536, afatinib and vincristine could all directly bind to RFC4 and form hydrogen bonds at low‐binding energy (Figure S15).

### 3.9 Identification of the potential biological function of RFC4 expression in HCC

Taken together, we found that RFC4 has good prognostic value and potential to be an immunotherapeutic target in hepatocellular carcinoma, therefore, we specifically analysed RFC4 in LIHC. First, 875 upregulated genes and 191 downregulated genes were identified when analysing differential genes in RFC4 high‐ and low‐expression samples in LIHC (Figure S16A). Supplementary data (Table S2) provided detailed gene information. The results of enrichment analysis showed that the upregulated differential genes were mainly associated with DNA replication, cell cycle, fatty acid degradation, valine, leucine and isoleucine degradation and Tryptophan metabolism, among others (Figure S16B,C). Samples with high expression of RFC4 had worse survival prognosis (Figure S16D), and Stage and RFC4 were found to be prognostic risk factors for LIHC by COX univariate and multivariate analysis (Figure S16E,F). Secondly, RFC4 somatic copy number variation was investigated by the CAMOIP database, and the analysis revealed that the mutation frequencies of genes including TP53, CYTNNB1, ALB and MUC4 were higher, and were more significant in the samples with high RFC4 expression (Figure S16G). To verify whether RFC4 has immunotherapeutic value in LIHC samples, we also calculated the immune cell differences between high‐ and low‐expression samples used the CIBERSORT algorithm, in which B cells, NK cell monocytes, macrophage M2 and mast cells had higher abundance in the RFC4 low‐expression samples, while Th cells, Treg, macrophage M0 and DC cells had higher abundance in RFC4 high‐expression samples (Figure S16H). Finally, we also analysed the correlation scores of immunotherapeutic indexes, and found that CAT scores, Th17 cells, Th2 cells were significantly different in RFC4 high‐expression and low‐expression samples (*p* < 0.0001, Figure S16I). Interestingly, the TCR Shannon score was not significantly different in RFC4 low expression samples. TCR Shannon score was not significant (ns, Figure S17A) and BCR Evenness score was more significant (*p* < 0.05, Figure S17B), whereas in RFC4 high expression samples, BCR Richness, BCR Shannon, Intratumor Heterogeneity, Aneuploidy Score and Homologous Recombination Defects had higher scores (Figure S17C–I). Most importantly RFC4 high expression samples had higher Proliferation and Wound Healing indices (*p* < 0.0001, Figure S17J,K).

### 3.10 Expression of RFC4 in LIHC and its impact on prognosis

**FIGURE 7:**
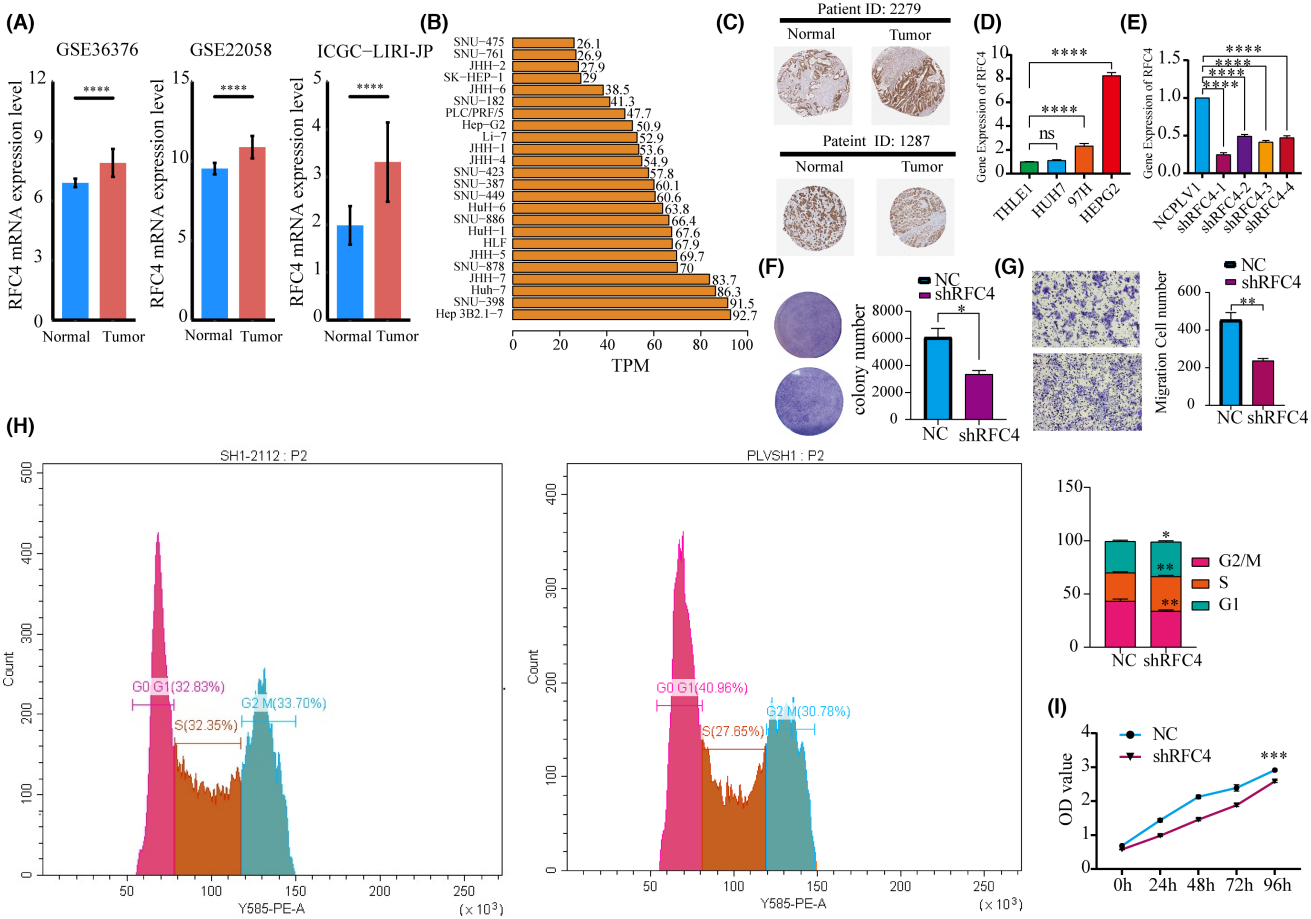
Expression and functional validation of RFC4 mRNA and protein in HCC. (A) RFC4 expression levels in normal and HCC from three datasets of the HCCDB database. (B) Expression of RFC4 in 24 HCC cell lines from the HPA database. (C) Protein expression of RFC4 in two hepatocellular carcinoma patients in the HPA database. (D) RFC4 mRNA expression in hcc cell lines. (E) Efficiency of sh‐RFC4 in HepG2 cells (F) Clone formation of HepG2 cell line. (G) Detection of cell migration and invasion using Transwell assay. (H) Proliferation ability of hcc cells detected by CCK8 (I) Proliferation ability of HCC cells detected by CCK8. *p < 0.05; **p < 0.01; ***p < 0.001; ****p < 0.0001.

To investigate the biological role of RFC4 in the growth of HCC cells, we validated the differential expression of RFC4 in cancer and adjacent tissues in three datasets of liver cancer from the HCCDB database (Figure 7A, *p* < 0.05). We then observed the expression of RFC4 in liver cancer cell lines from the HPA database and found that it was highly expressed in Huh7 and Hep3B (Figure 7B). We observed a high distribution of RFC4 in pathological tissues (Figure 7C) and found differential high expression in HepG2 and 97H (Figure 7D). HepG2 cells were successfully silenced with RFC4 shRNA (Figure 7E). Subsequently, we evaluated cell growth by measuring clone formation and invasion. The results showed that after RFC4 silencing, the clone formation and invasion of HepG2 cells significantly reduced (Figure 7F,G, *p* < 0.05). It was verified that RFC4 has a significant effect on the cell cycle of HCC cells (Figure 7H). Additionally, the Transwell assay demonstrated that knocking down RFC4 significantly decreased the proliferation of HCC cells (Figure 7I).

## 4 DISCUSSION

RFC4 is a protein‐coding gene that encodes a 37 kDa subunit. This subunit forms a core complex with 36 and 40 kDa subunits, which has DNA‐dependent ATPase activity. This activity catalyses the binding of proliferating cell nuclear antigen to DNA, induces DNA polymerase to the end of RNA primers, and then participates in DNA synthesis by consuming ATP to provide energy.^21^, ^22^ RFC4 differential expression is also shown to be higher in many human malignant tumours, including oral squamous cell carcinoma^23^ and colorectal cancer.^12^ RFC4 is a potential target in the progression of nasopharyngeal carcinoma. Additionally, RFC4 knockout can induce G2/M cell cycle arrest and inhibit nasopharyngeal carcinoma cell proliferation. Overexpression of HOXA10 can alleviate RFC4‐induced cell proliferation silencing, colony formation inhibition and cell cycle arrest.^24^ The abnormal expression of RFC4 in different tumours is important for the occurrence and personalized treatment of tumours, which can increase the potential targets for successful cancer treatment. Therefore, we conducted systematic pan cancer research on RFC4 based on multiple databases. Abnormal expression of RFC4 was found in different types of cancer used TCGA and GTEx data. The analysis of TCGA genome and survival data can provide valuable guidance for clinical research. Our study reveals that RFC4 is upregulated in various tumours including GBM, CESC, LUAD, COAD, BRCA, HNSC, KIRC, LUSC and LIHC. The evidence from HCC samples suggests that elevated levels of RFC4 in tumour tissue are related to tumour growth and prognosis, which is consistent with our research findings.^25^ The purpose of our study was to analyse the relationship between RFC4 and survival rate in order to better understand its role in clinical risk stratification. Our findings indicate that overexpression of RFC4 is associated with poorer prognosis in patients with LUAD, ACC, LIHC, KICH, KIRC, LGG and KIRP (*p* < 0.05) based on survival studies of OS, DSS, DFI and PFI. While previous studies have suggested that RFC4 affects the development of HCC, further research is needed to fully understand its role in these cancers.^26^ Abnormal DNA methylation and differential splicing can cause dysregulation of cancer gene expression, which affects cancer development and progression.^27^, ^28^, ^29^, ^30^, ^31^, ^32^, ^33^ Our research delved into the relationship between methylation, alternative splicing and RFC4 expression. We found differential methylation of RFC4 in various cancers. Differences in methylation patterns were observed among various tumours, which may be attributed to inherent differences. A statistically significant negative correlation between RFC4 expression and methylation was found in most tumours, indicating that methylation‐mediated RFC4 gene expression plays a crucial role in tumour development. Alternative splicing of RFC4 was detected in different cancers. Significant differences in the level and prognostic value of variable splicing were found among different tumours. This suggests that the mechanism of tumour occurrence can be explored and diagnostic predictions can be made based on the alternative splicing of RFC4.

The tumour microenvironment is a complex composition of various cellular components.^15^, ^34^ Our analysis of single‐cell sequencing data from different tumours revealed that RFC4 is highly expressed in Tprolif cells and tumour cells. This suggests that RFC4 may activate T cells to inhibit tumour growth, while also being an essential gene for tumour cell growth. Normally, the immune system can recognize and eliminate tumour cells in the TME. However, tumour cells can evade the immune system's killing mechanisms in various ways.^35^, ^36^ We collected common immune checkpoint genes and immune regulatory factors and estimated their correlation with RFC4 expression. In cancer patients, most immune checkpoint genes and immune regulatory factors showed a strong correlation with RFC4. Based on the results of three algorithms, TIMER, MCPCOUNTER and CIBERSORT, we found a significant correlation between the expression level of RFC4 in tumours such as KIRC, LUAD and LIHC and the infiltration degree of CD4 + T cells, CD8 + T cells, B cells, macrophages and dendritic cells. It has been discovered that TMB levels can impact the generation of immunogenic peptides, which in turn affects the patient's response to immune checkpoint inhibitors (ICI).^37^, ^38^ Therefore, TMB can serve as a reliable indicator of the effectiveness of ICI, and tumours with higher TMB are more likely to respond favourably to cancer immunotherapy.^39^ Therefore, TMB can serve as a reliable indicator of the effectiveness of ICI, and tumours with higher TMB are more likely to respond favourably to cancer immunotherapy. Several studies have reported a correlation between higher TMB and ICI efficacy, indicating that TMB could serve as a reliable predictive biomarker.^38^, ^40^, ^41^, ^42^ This study examines the correlation between RFC4 expression and TMB and MSI levels based on TCGA data. The results indicate that RFC4 expression is correlated with TMB and MSI levels in BLCA, COAD, KICH, KIRC, LGG, LUAD, PRAD, READ, SKCM, STAD and UCEC. Therefore, detecting the expression level of RFC4 can be used to evaluate the effectiveness of immunotherapy in the future. Additionally, combining targeted therapy of RFC4 with traditional immunotherapy can improve its effic. When dysfunction occurs in CTLs, they promote immune escape of the tumour, leading to tumour growth, invasion, metastasis and treatment resistance.^43^, ^44^ According to Miao 2018_ICB and Riaz 2017_PD1 queue, high expression of RFC4 affects the efficacy of PD1 blockade in KIRC and SKCM patients, and reduces overall survival and progression‐free survival. High expression of RFC4 can also affect the immune response of UVM. These findings indicate that RFC4 is involved in tumour immune evasion. In the cohort of cancer patients treated with BI‐2536, RFC4 expression was found to be high in responders with breast cancer. Conversely, in patients treated with CD‐327, RFC4 expression was high in non‐responders with breast, liver and lung cancer, but was high in responders with gastric cancer and head and neck cancer. In the cohort of cancer patients treated with BI‐2536, RFC4 expression was found to be high in responders with breast cancer. Similarly, in non‐responders treated with vincristine, RFC4 expression was high in brain, endometrial, liver and lung cancers, as well as in gastric cancer in RFC4 responders. The level of RFC4 expression was found to be directly proportional to the resistance of anti‐tumour drugs such as vinorelbine. Further research is necessary to establish the relationship between RFC4 and chemotherapy resistance. Further research is necessary to establish the relationship between RFC4 and chemotherapy resistance. Studies have demonstrated the formation of a positive feedback loop between high levels of RFC4 and NICD1, resulting in sustained overactivation of Notch signal transduction. This leads to tumorigenicity and metastasis of NSCLC, as well as resistance of NSCLC cells to the drug DAPT, which is used for NICD1 synthesis in clinical trials.^45^ Therefore, it is important to investigate the correlation between RFC4 and chemotherapy resistance in more detail.

We investigated the role of RFC4 in cancer using molecular biology methods. RFC4 is involved in cancer cell cycle and growth pathways and has a high immune infiltration and proliferation score. Immunohistochemistry and qPCR confirmed that RFC4 is upregulated in HCC tissues. Edu staining further confirmed that RFC4 promotes HCC cell proliferation. Flow cytometry sorting revealed that knocking down RFC4 led to an increase in the proportion of G0/G1 phase cells, confirming the proliferative effect of RFC4. Transwell experiments further confirmed that RFC4 can promote the invasion of cancer cells. These results validate the accuracy and reliability of the pan‐cancer bioinformatics analysis, and we plan to conduct similar molecular biology verifications in other types of cancer in the future. However, this study has some limitations despite exploring and integrating information from multiple databases. Bioinformatics analysis provides important information on the role of RFC4 in malignant tumours. To validate our results and improve treatment effectiveness, further in vitro and in vivo biological experiments are necessary. Although RFC4 expression is associated with immune and clinical survival in human malignant tumours, it is unclear whether RFC4 affects clinical survival through immune pathways. Our pan‐cancer comprehensive analysis demonstrates the characteristics of RFC4 in cancer tissues and identifies it as an important prognostic biomarker for certain types of cancer. The results of this study show that RFC4 levels are associated with cancer immunotherapy. Prospective studies that focus on RFC4 expression and the tumour immune environment will help provide conclusive answers, facilitating the development of immune‐based anti‐cancer therapies in the future.

## 5 CONCLUSIONS

The study suggests that RFC4 could serve as a potential biomarker for diagnosing and predicting the prognosis of certain tumour, including HCC. It is related to immune cell infiltration in the tumour microenvironment and may be a potential target for immunotherapy in cancers such as HCC, LUAD and KIRP. RFC4 has been identified as a regulatory factor for the expression of genes related to HCC cell proliferation and invasion. These findings provide a foundation for further research and the development of targeted therapies for HCC.

## AUTHOR CONTRIBUTIONS

**Lei Yu:** Formal analysis (lead); methodology (lead); visualization (lead); writing – original draft (lead). **Jing Li:** Methodology (supporting); validation (equal). **Mingyang Zhang:** Validation (equal); writing – original draft (supporting). **Yu Li:** Investigation (supporting); validation (equal). **Jing Bai:** Validation (equal). **Pengxia Liu:** Conceptualization (equal); project administration (lead). **Jia Yan:** Conceptualization (equal); writing – review and editing (equal). **Changshan Wang:** Conceptualization (equal); resources (lead); supervision (lead); writing – review and editing (equal).

## CONFLICT OF INTEREST STATEMENT

The authors declare that the research was conducted in the absence of any commercial or financial relationships that could be construed as a potential conflict of interest.

## Supporting information


Figures S1–S17.

Table S1.

Table S2.

## Data Availability

Online repositories contain the datasets mentioned in this study. The names of the repository/repositories and accession number(s) can be found in the article/Supplementary Material.
