# Supplementary material for: Identification of RFC4 as a potential biomarker for pan‐cancer involving prognosis, tumour immune microenvironment and drugs
Source: J Cell Mol Med. 2024 Jun 21;28(12):e18478. doi: 10.1111/jcmm.18478 (PMC11190950; doi:10.1111/jcmm.18478)
Supplement: Supplementary file 1 — Figures S1–S17. [file JCMM-28-e18478-s001.pdf]

Supplementary materials

Supplementary Figures

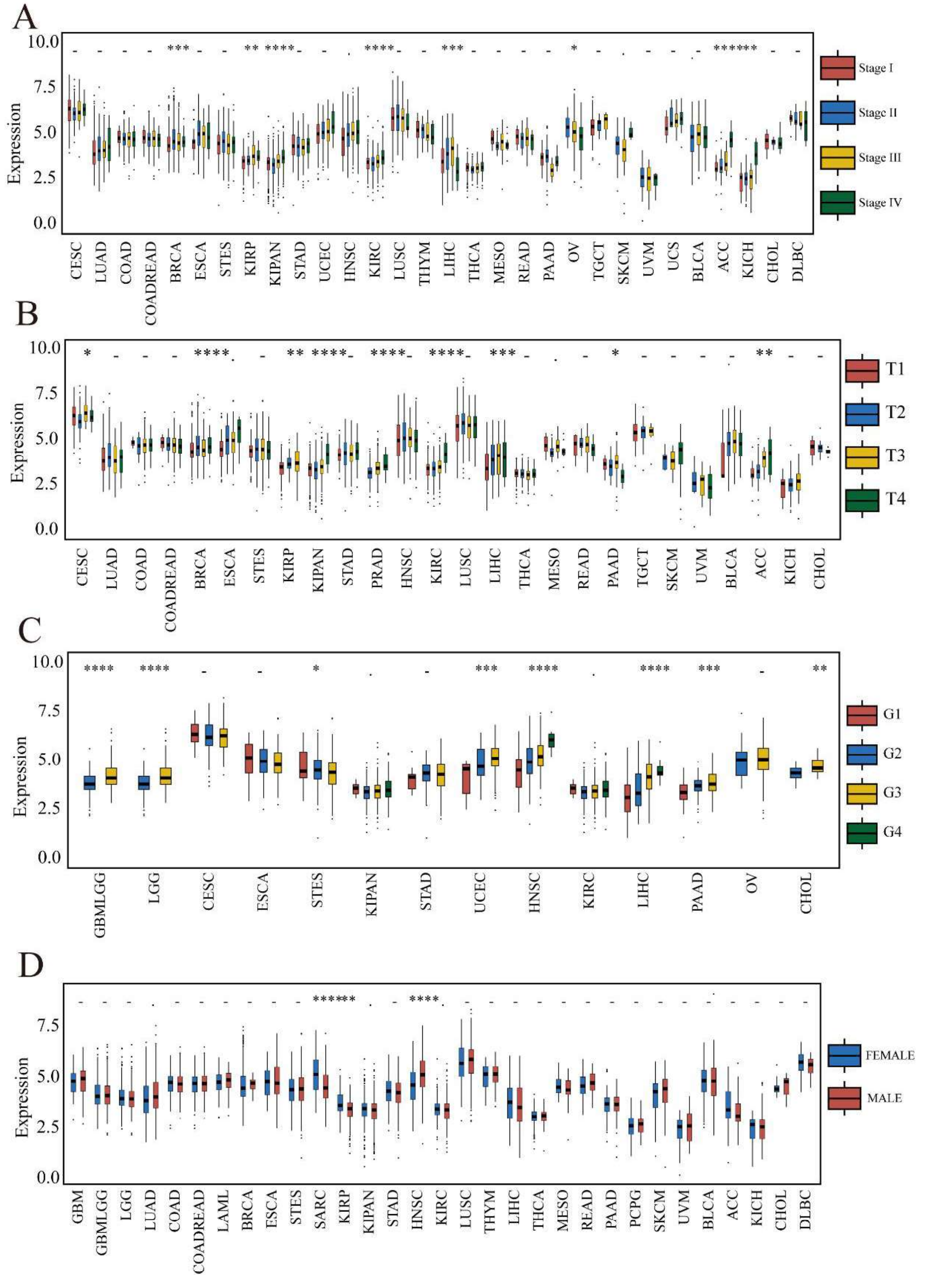

Supplementary Figure 1： (A) The correlation between RFC4 expression and the pathological stages of cancers. (B). The correlation between RFC4 expression and the pathological T of cancers. (C) The correlation between RFC4 expression and the pathological Grade of cancers. (D). The correlation between RFC4 expression and the pathological sex of cancers. *$p<0.05$; **$p<0.01$; ***$p<0.001$; ****$p<0.0001$.

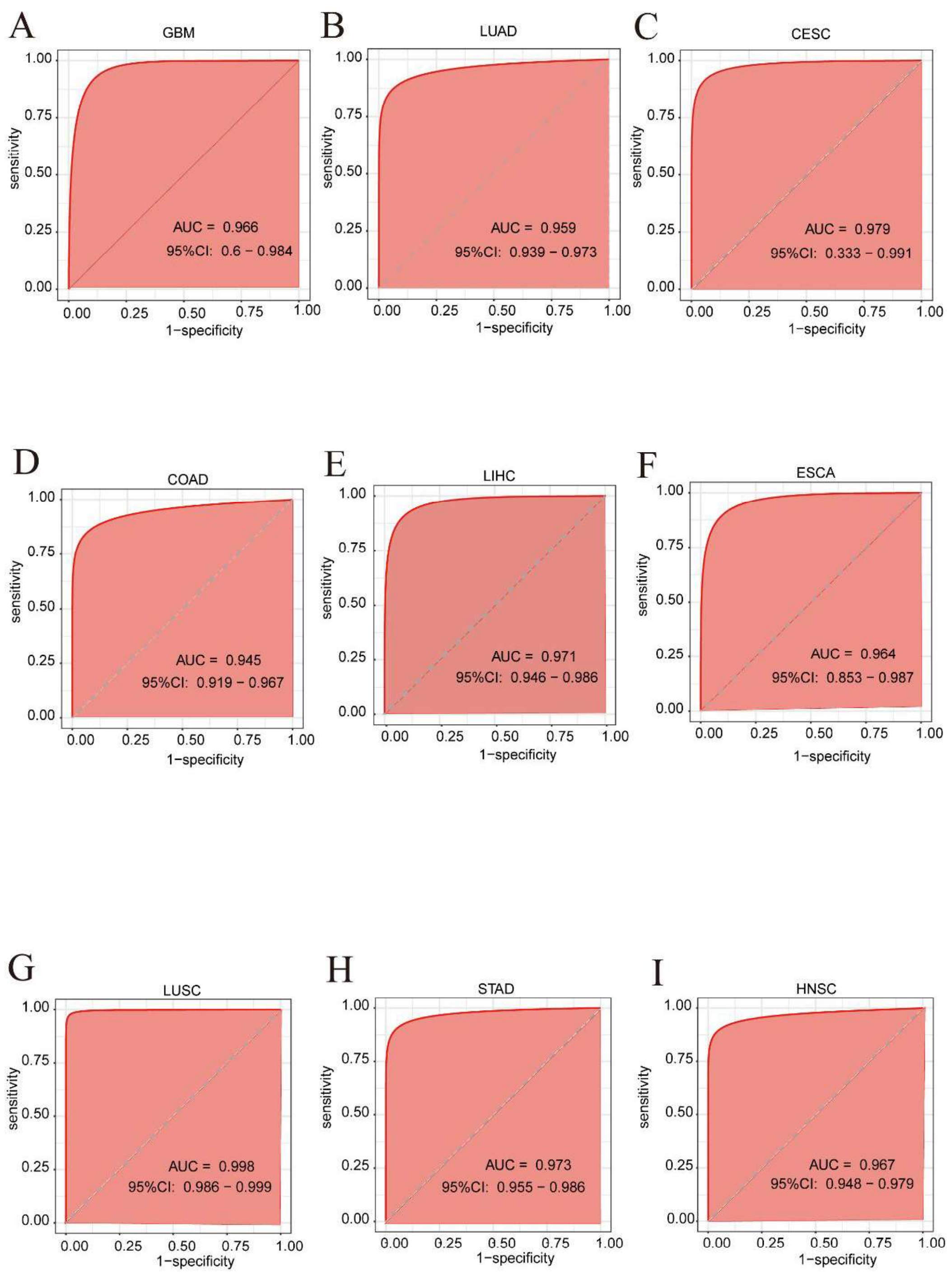

Supplementary Figure 2: The ROC curve of RFC4 in cancer. (A). GBM. (B). LUAD. (C). CESC. (D) COAD. (E). LIHC. (F). ESCA. (G). LUSC. (H). STAD. (I). HNSC

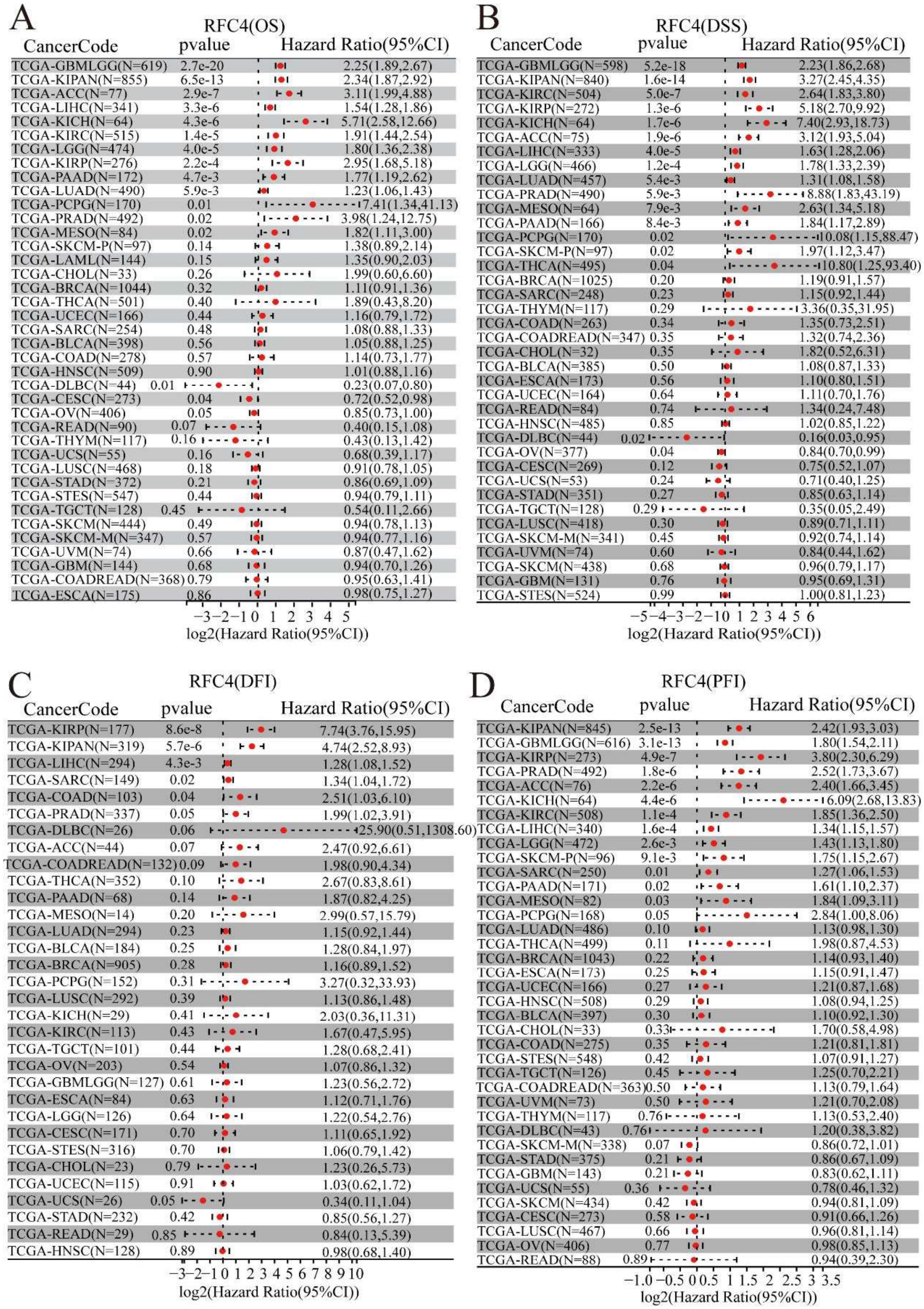

Supplementary Figure 3: Prognosis analysis of RFC4 in cancers from the TCGA database using the Sangerbox website tool. (A). Overall survival. (B). Disease-specific survival. (C). Disease-free interval. (D) Progression-free interval.

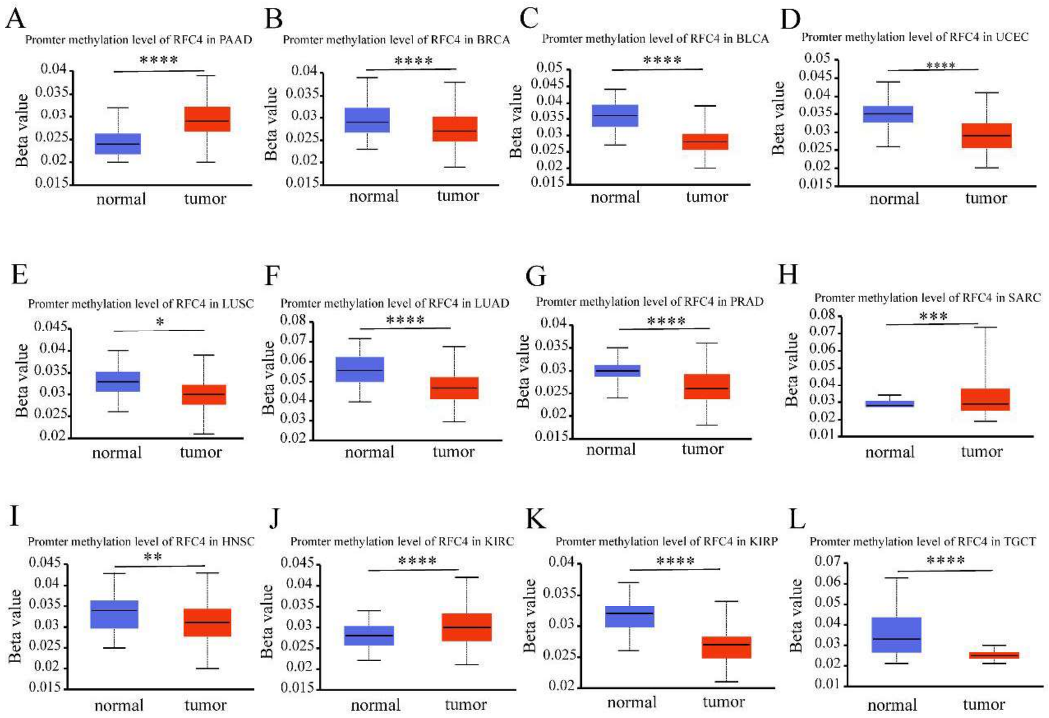

Supplementary Figure 4: Differences in DNA methylation levels between tumor and normal samples in pan-cancer. (A)PAAD. (B). BRCA. (C). BLCA. (D) UCEC. (E) LUSC. (F) LUAD (G). PRAD. (H). SARC. (I) HNSC. (J) KIRC. (K) KIRP. (L)TGCT *p<0.05; **p<0.01; ***p<0.001; ****p<0.0001.

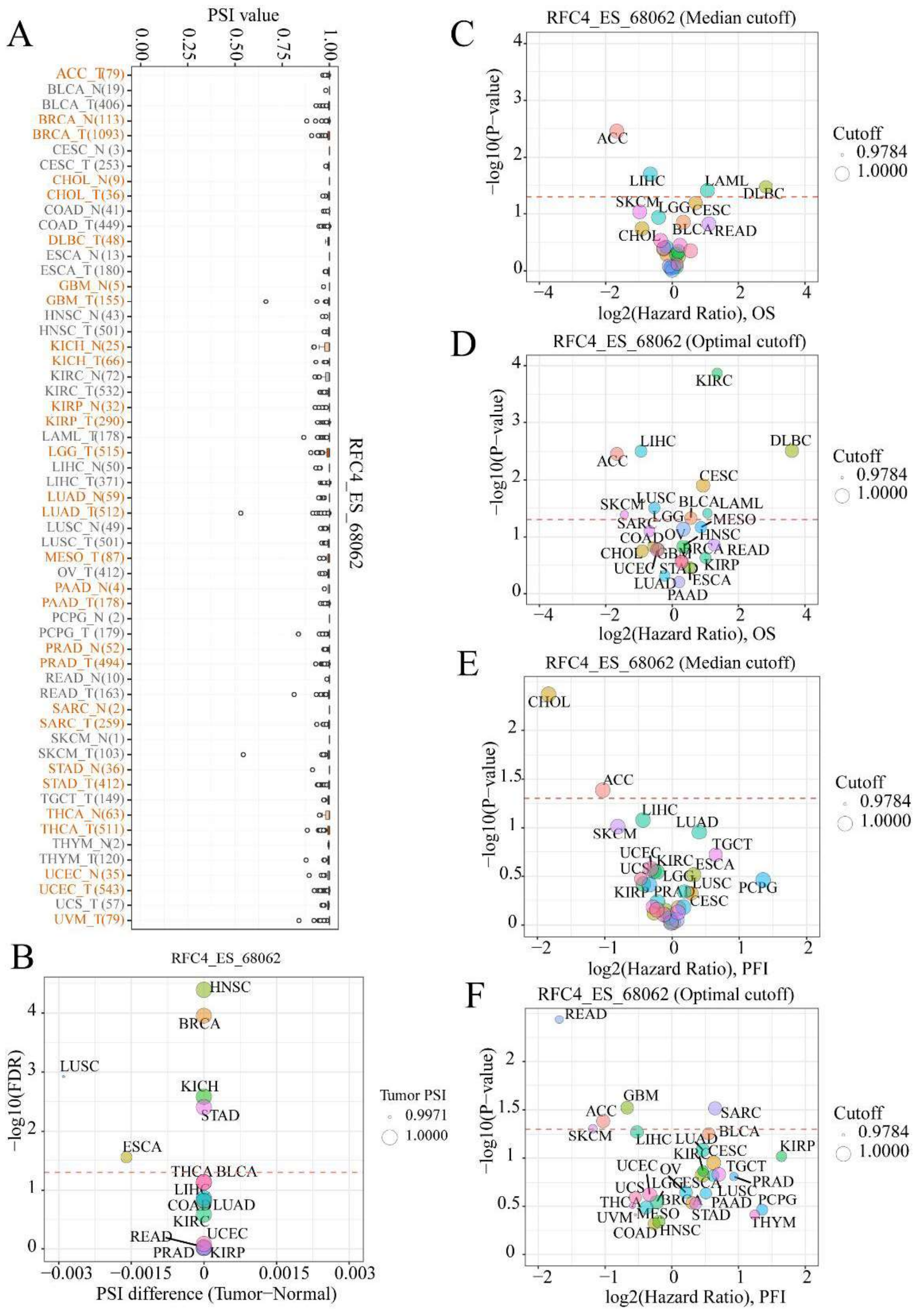

Supplementary Figure 5: Expression level and prognostic value of RFC4_ES_68062 differential splicing in pan-cancer

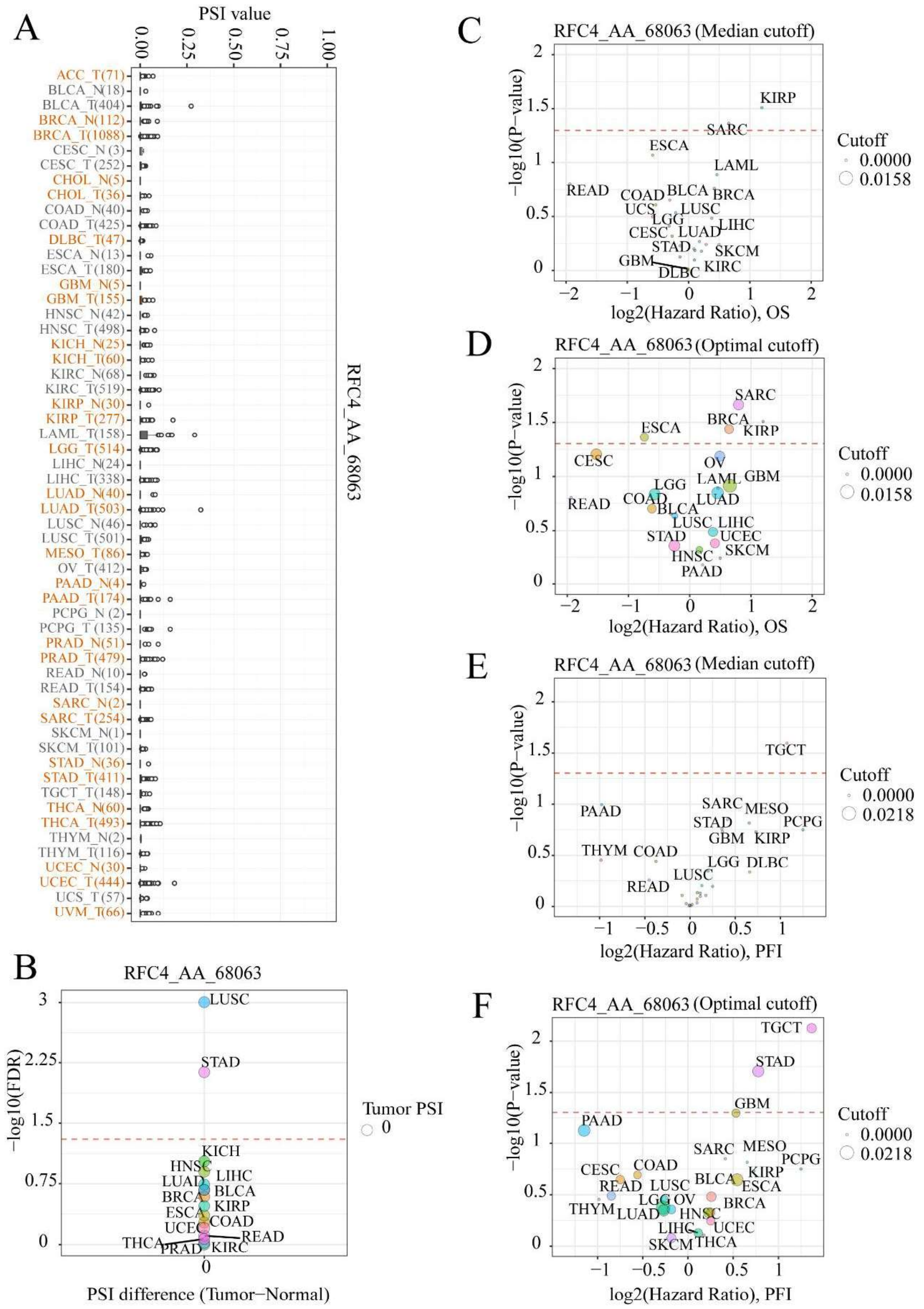

Supplementary Figure 6: Expression level and prognostic value of RFC4_ES_68063 differential splicing in pan-cancer.

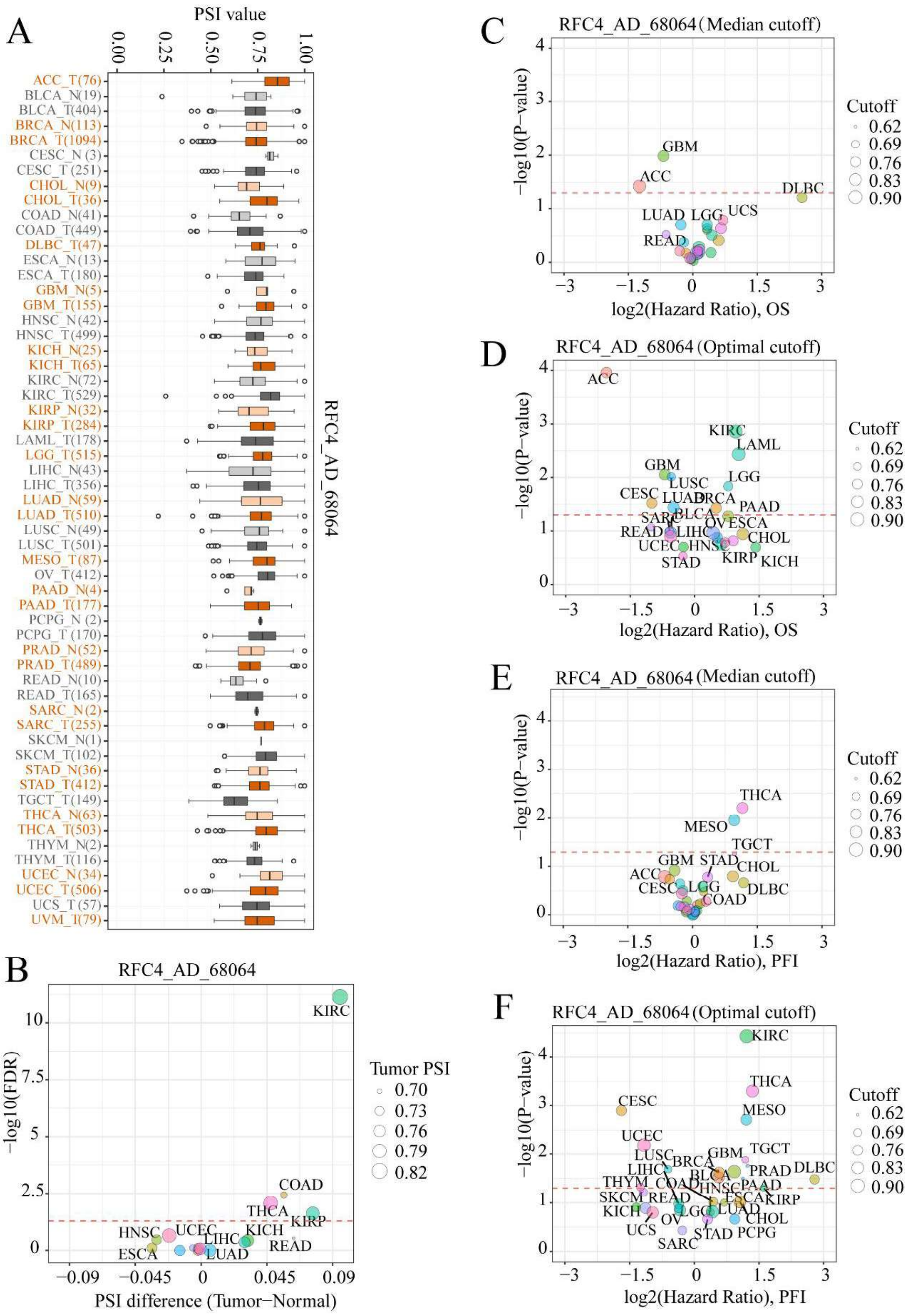

Supplementary Figure 7: Expression level and prognostic value of RFC4_ES_68064 differential splicing in pan-cancer.

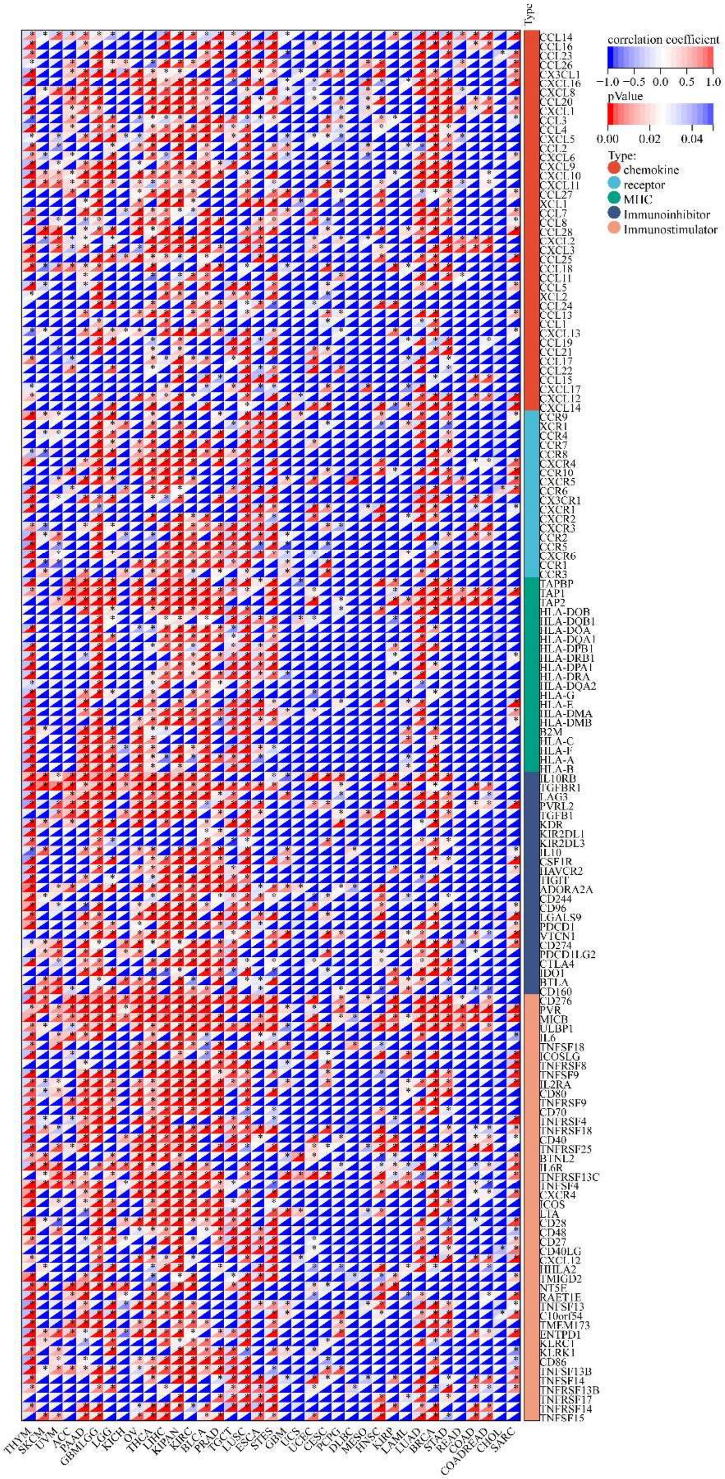

Supplementary Figure 8: The clustering heatmap shows the correlation analysis between the expression levels of RFC4 and immune checkpoint genes in cancer. Red indicates positive correlation, while blue indicates negative correlation.*p<0.05; **p<0.01; ***p<0.001; ****p<0.0001.

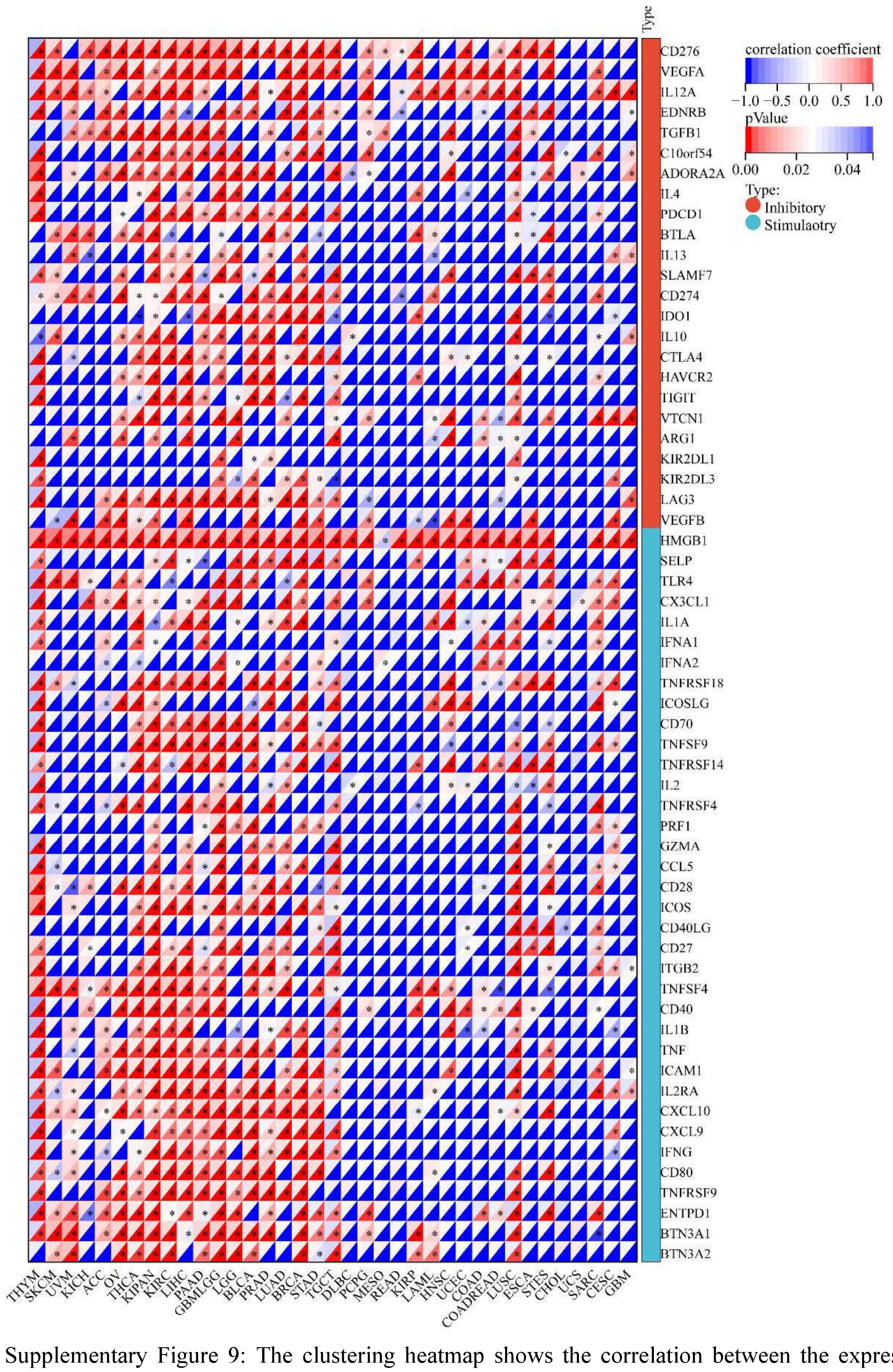

Supplementary Figure 9: The clustering heatmap shows the correlation between the expression levels of RFC4 and immune regulatory factors in cancer. Red indicates positive correlation, while blue indicates negative correlation.*p<0.05; **p<0.01; ***p<0.001; ****p<0.0001.

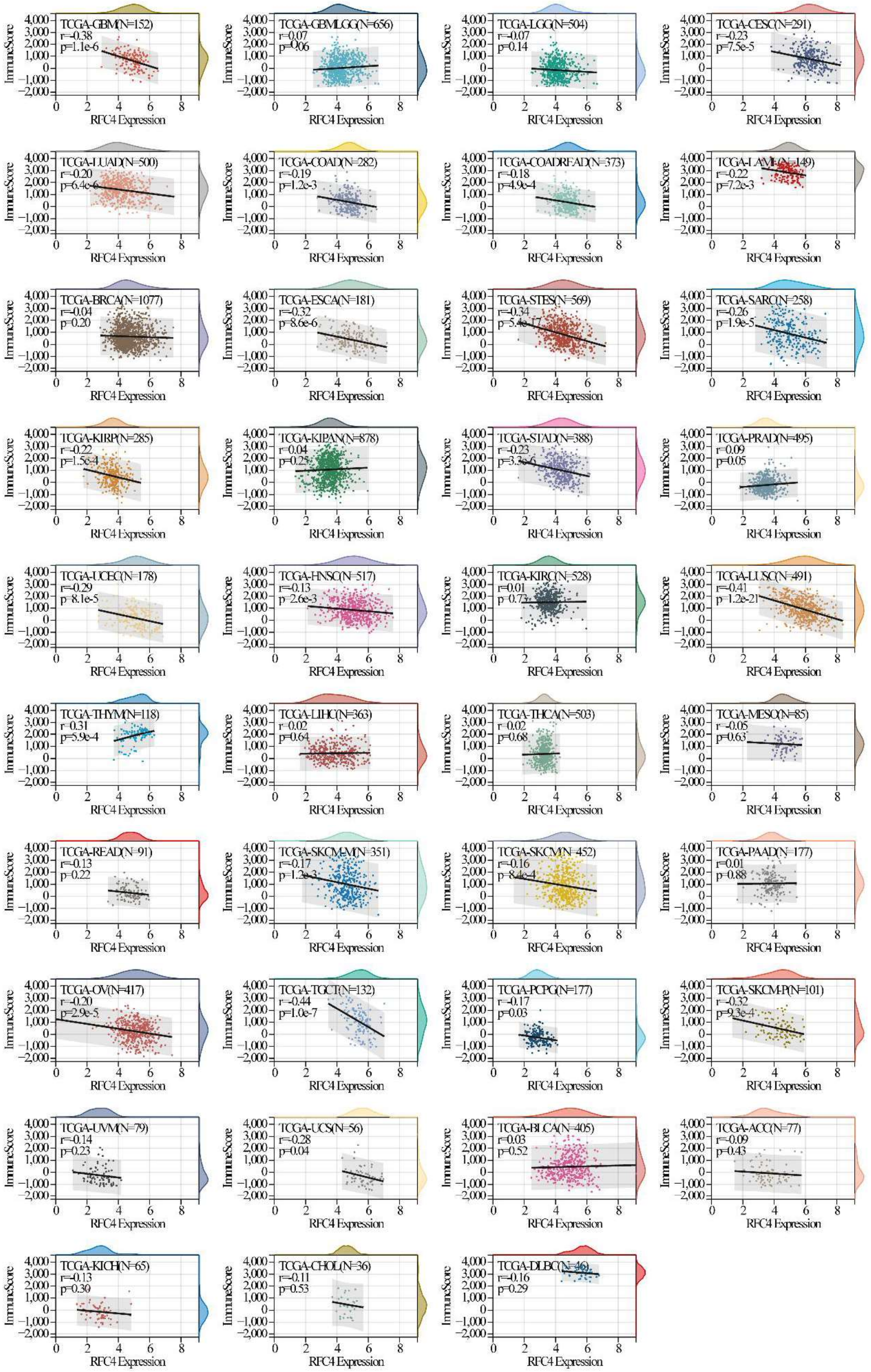

Supplementary Figure 10：Correlation of RFC4 expression with ImmuneScore.

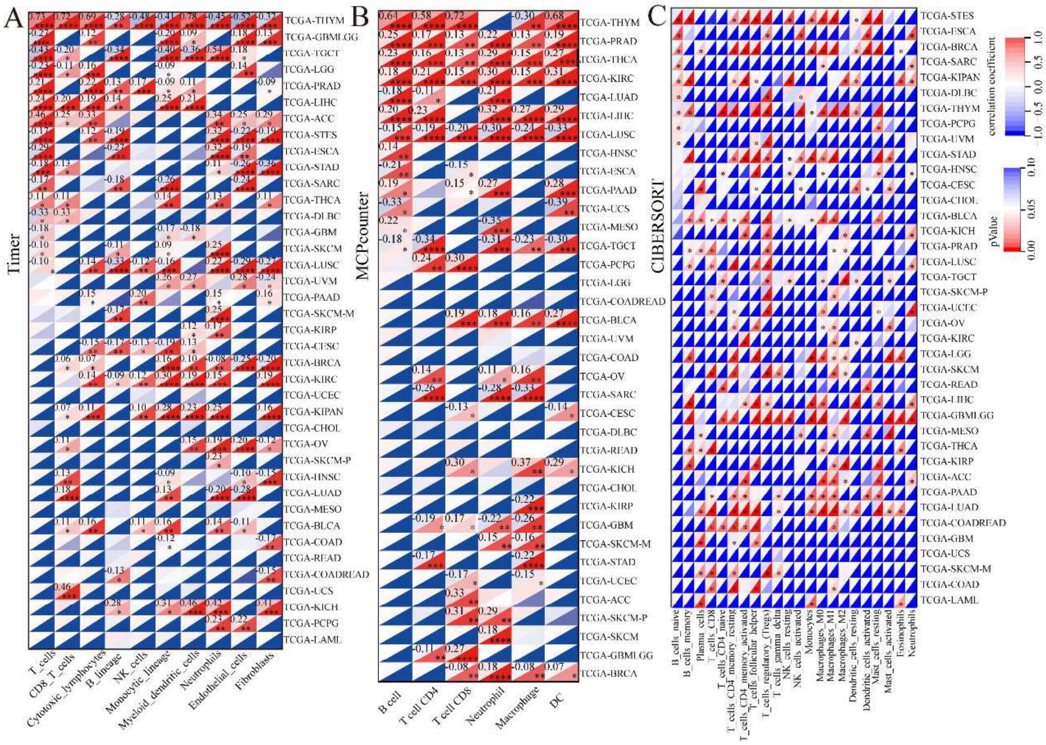

Supplementary Figure 11: Correlation of RFC4 with the level of immune infiltrating cells in tumor (A) Correlation of RFC4 with the level of immune infiltration in cancers analyzed by the Timer algorithm. (B). Correlation of RFC4 with the level of immune infiltration in cancers analyzed by the MCPcounter algorithm. (C). Correlation of RFC4 with the level of immune infiltration in cancers analyzed by the CIBERSORT algorithm. *p<0.05; **p<0.01; ***p<0.001; ****p<0.0001.

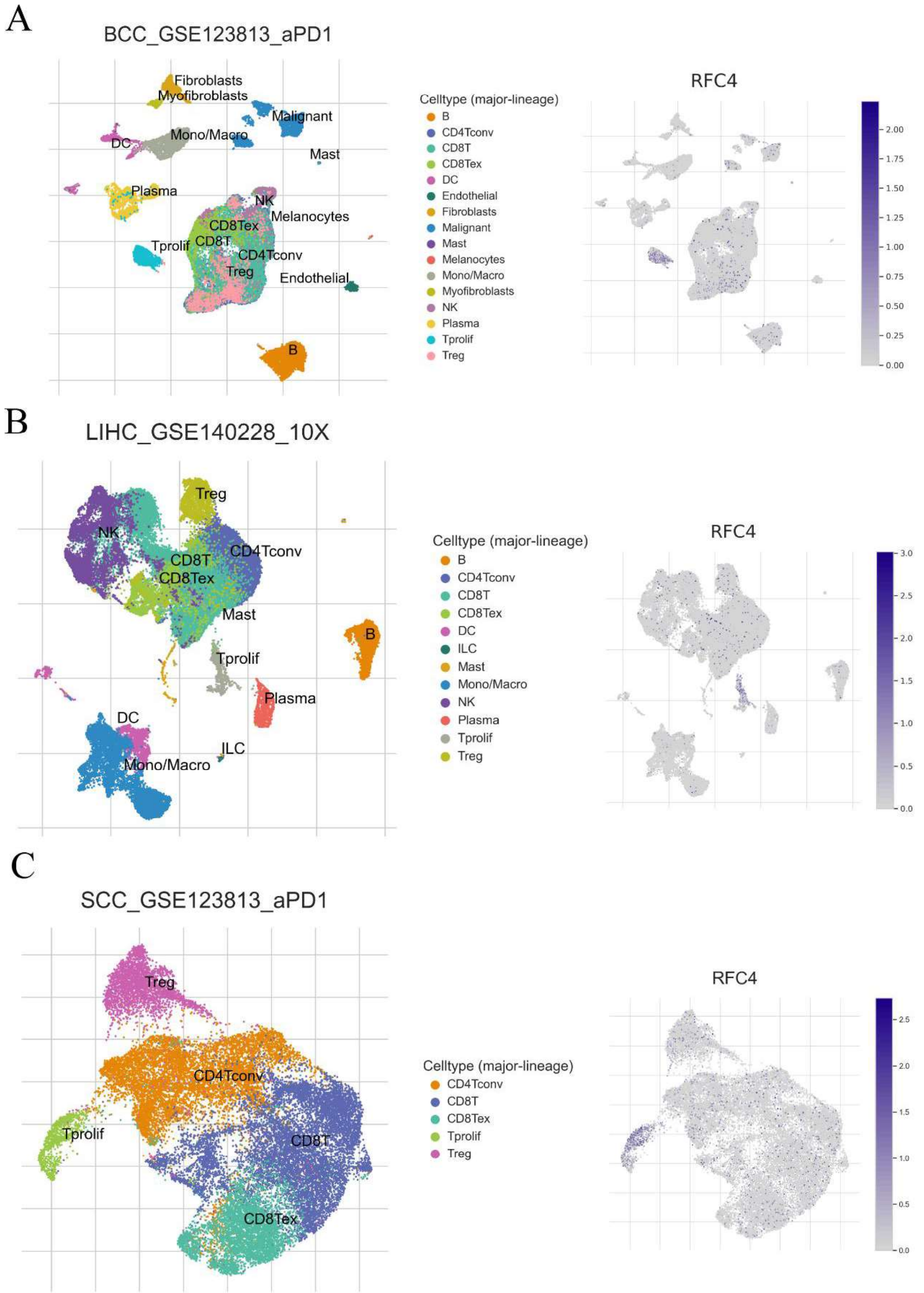

Supplementary Figure 12: Single-cell expression analysis of RFC4 in tumor tissues. Umap plots displaying the clustering of different cell types and RFC4 expression level in BCC(A), LIHC(B) and SCC(C).

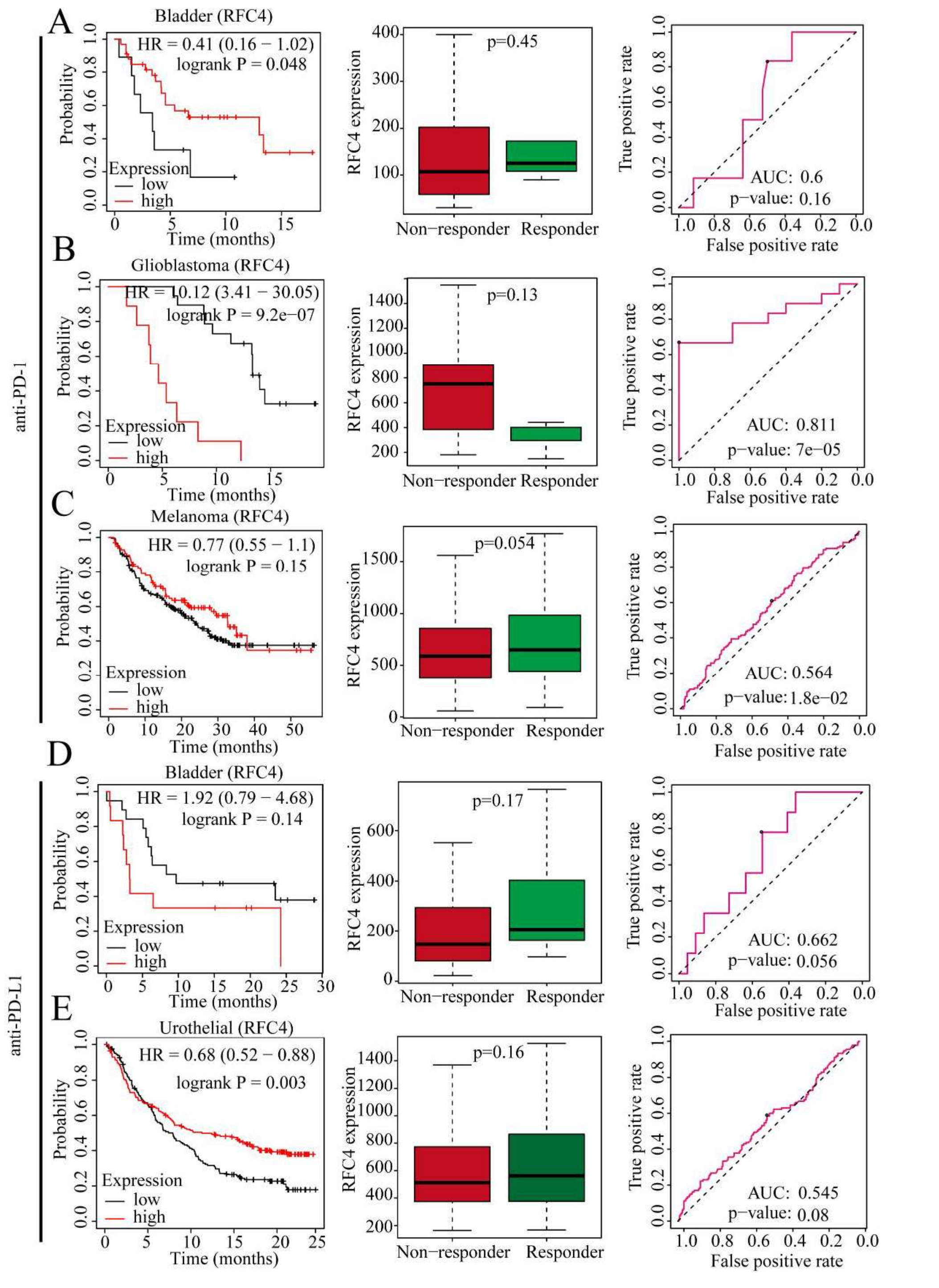

Supplementary Figure 13： Response and drug sensitivity analysis of RFC4 to immunotherapy. (A)The relationship between RFC4 expression level and anti-PD-1 in Bladder (B) The relationship between RFC4 expression level and anti-PD-1 in Glioblastoma (C) The relationship between RFC4 expression level and anti-PD-1 in Melanoma. (D) The relationship between RFC4 expression level and anti-PD-L1 in Bladder (E) The relationship between RFC4 expression level and anti-PD-L1 in Urothelial. The ROC curve reflects the predictive effect of RFC4 levels on immunotherapy response.

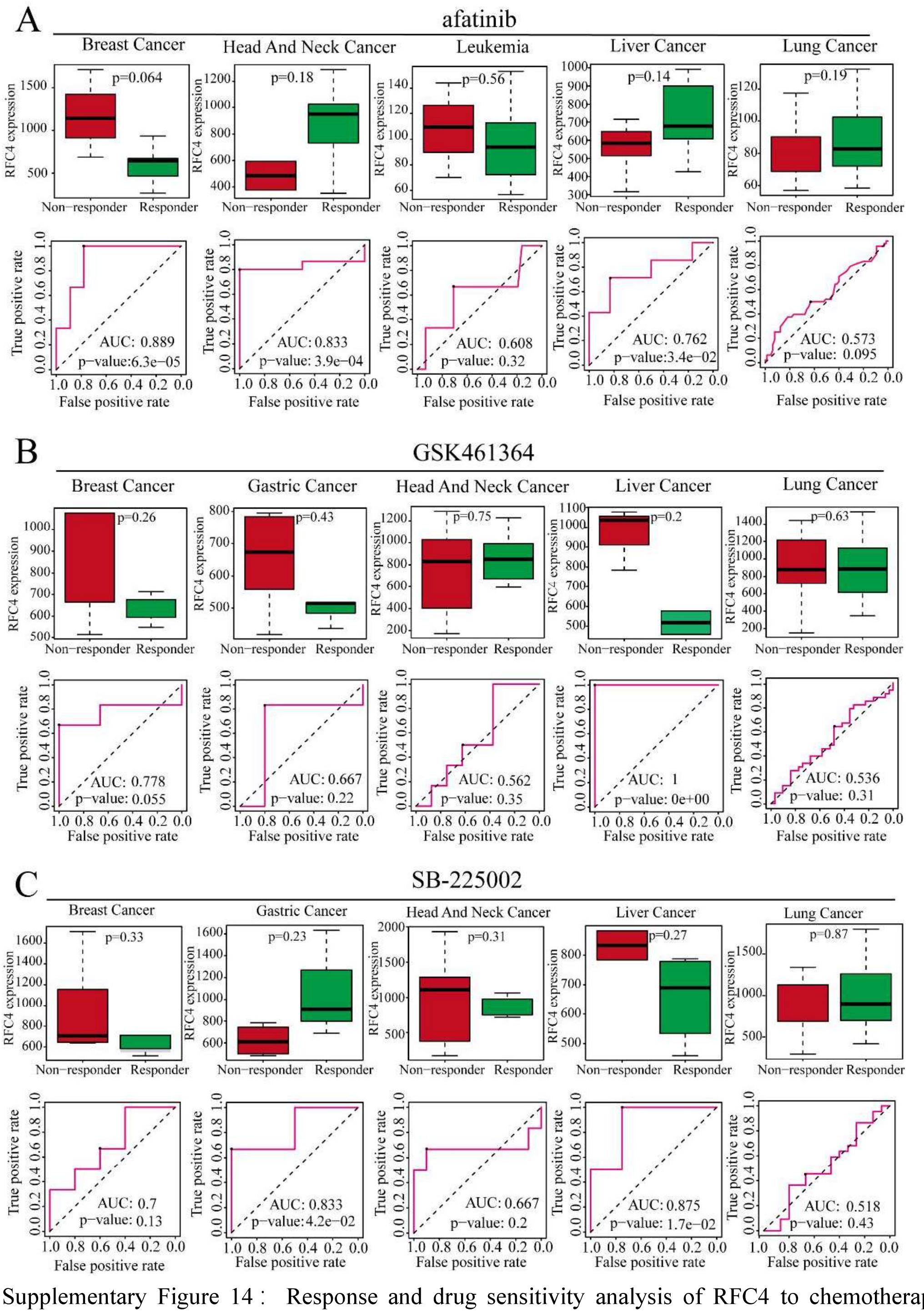

Supplementary Figure 14： Response and drug sensitivity analysis of RFC4 to chemotherapy. (A)The relationship between RFC4 expression level and afatinib in 5 types of cancer, (B) the relationship between RFC4 expression level and GSK461364 in 5 types of cancer, and (C) the relationship between RFC4 expression level and SB-225002 in 5 types of cancer. The ROC curve reflects the predictive effect of RFC4 levels on chemotherapy response.

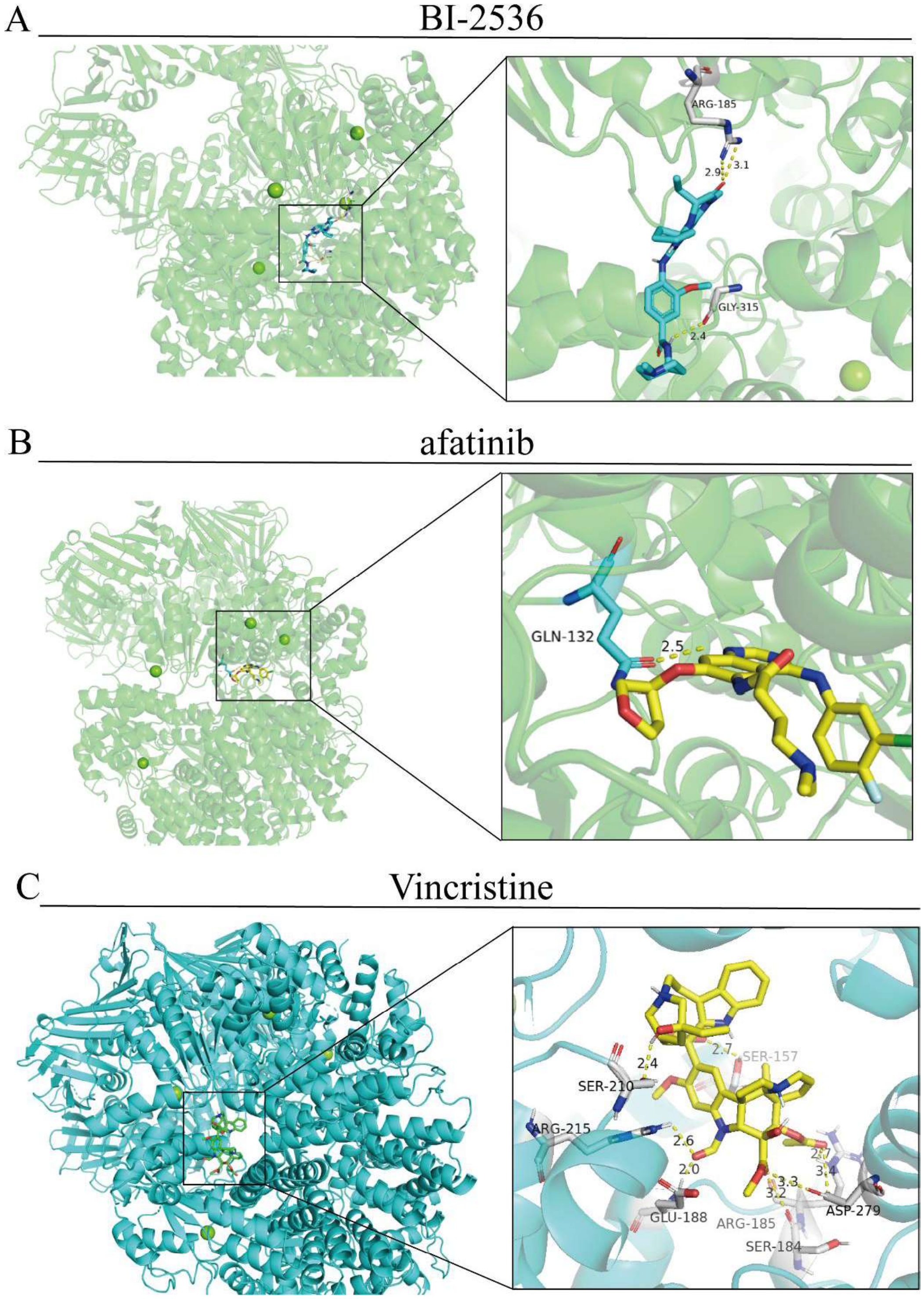

Supplementary Figure 15： Docking of RFC4 with drug molecules (A) BI-2536. (B) afatinib. (C) Vincristine

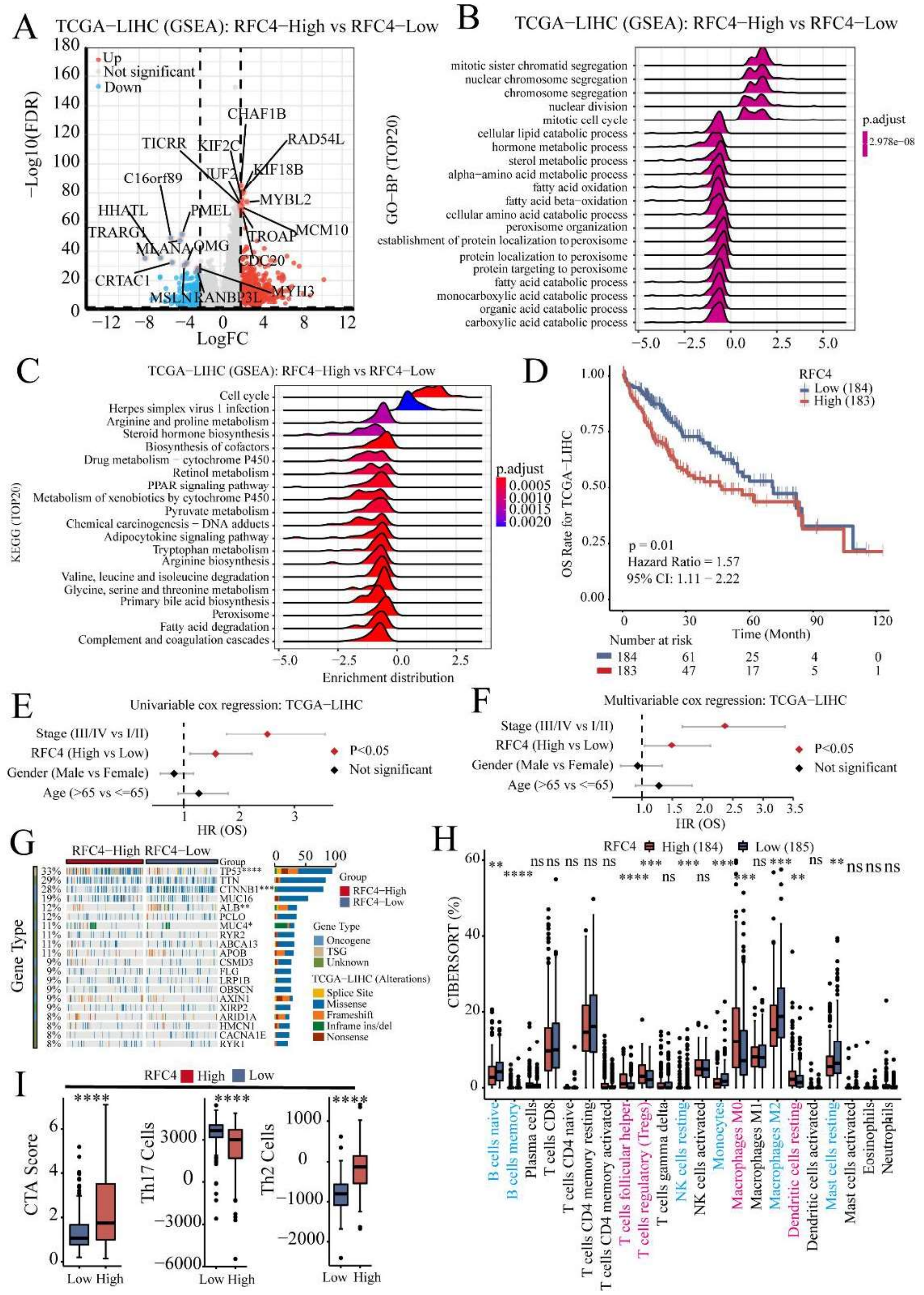

Supplementary Figure 16: Potential biological functions of RFC4 in HCC. (A) Volcano plot of differential genes in RFC4 high and low expression samples. (B) Enrichment analysis of differential genes (GO). (C) Enrichment analysis of differential genes (KEGG). (D) Log-rank test for RFC4 expression in LIHC. (E) Univariate COX regression analysis of RFC4 in LIHC samples (F) Multivariate COX regression analysis of RFC4 in LIHC samples. (G) The top 20 mutated genes and their frequency differences based on the high and low expression levels of RFC4. (H) Relative infiltration abundance of 22 immune cell types in high and low RFC4 samples. (I) Differences in CTA Score, Th17 Cells, and Th2 Cells between high and low RFC4 samples *p<0.05; **p<0.01; ***p<0.001; ****p<0.0001.

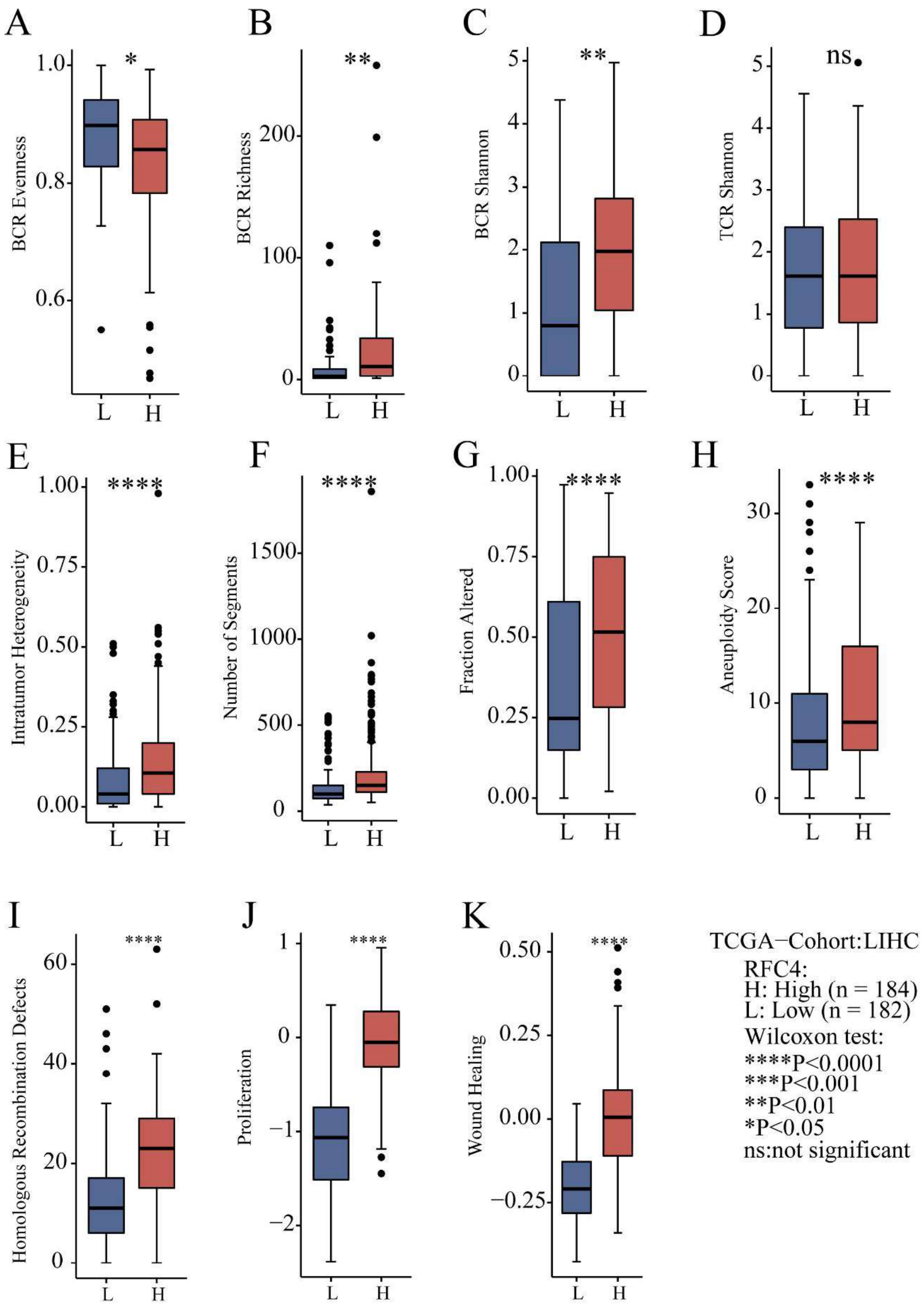

Supplementary Figure 17: Immunity and bioactivity scores of RFC4 in high and low expression samples (A) TCR Shannon; (B) BCR Richness; (C) BCR Shannon; (D) BCR Evenness; (E) Intratumor Heterogeneity; (F) Number of Segments; (G) Fraction Altered; (H) Aneuploidy Score; (I) Homologous Recombination Defects, (I) Proliferation; (I) Wound Healing. NS>0.05; *p<0.05; **p<0.01; ***p<0.001; ****p<0.0001.

Supplementary Tables

Supplementary Table 1. Methylation prognosis information of RFC4.

Supplementary Table 2. Differential genes in high and low expression groups of RFC4 in HCC.
